# Identification of fatty acid metabolism–related molecular subtype biomarkers and their correlation with immune checkpoints in cutaneous melanoma

**DOI:** 10.3389/fimmu.2022.967277

**Published:** 2022-11-18

**Authors:** Yujian Xu, Youbai Chen, Weiqian Jiang, Xiangye Yin, Dongsheng Chen, Yuan Chi, Yuting Wang, Julei Zhang, Qixu Zhang, Yan Han

**Affiliations:** ^1^ Department of Plastic and Reconstructive Surgery, The First Medical Center of Chinese PLA General Hospital, Beijing, China; ^2^ Department of Plastic Surgery, The University of Texas MD Anderson Cancer Center, Houston, TX, United States

**Keywords:** cutaneous melanoma, tumor metabolism, fatty acid metabolism, immune infiltration, immune checkpoint, prognosis, tumor microenvironment

## Abstract

**Purpose:**

Fatty acid metabolism (FAM) affects the immune phenotype in a metabolically dynamic tumor microenvironment (TME), but the use of FAM-related genes (FAMGs) to predict the prognosis and immunotherapy response of cutaneous melanoma (CM) patients has not been investigated. In this study, we aimed to construct FAM molecular subtypes and identify key prognostic biomarkers in CM.

**Methods:**

We used a CM dataset in The Cancer Genome Atlas (TCGA) to construct FAM molecular subtypes. We performed Kaplan–Meier (K-M) analysis, gene set enrichment analysis (GSEA), and TME analysis to assess differences in the prognosis and immune phenotype between subtypes. We used weighted gene co-expression network analysis (WGCNA) to identify key biomarkers that regulate tumor metabolism and immunity between the subtypes. We compared overall survival (OS), progression-free survival (PFS), and disease-specific survival (DSS) between CM patients with high or low biomarker expression. We applied univariable and multivariable Cox analyses to verify the independent prognostic value of the FAM biomarkers. We used GSEA and TME analysis to investigate the immune-related regulation mechanism of the FAM subtype biomarker. We evaluated the immune checkpoint inhibition (ICI) response and chemotherapy sensitivity between CM patients with high or low biomarker expression. We performed real-time fluorescent quantitative PCR (qRT-PCR) and semi-quantitative analysis of the immunohistochemical (IHC) data from the Human Protein Atlas to evaluate the mRNA and protein expression levels of the FAM biomarkers in CM.

**Results:**

We identified 2 FAM molecular subtypes (cluster 1 and cluster 2). K-M analysis showed that cluster 2 had better OS and PFS than cluster 1 did. GSEA showed that, compared with cluster 1, cluster 2 had significantly upregulated immune response pathways. The TME analysis indicated that immune cell subpopulations and immune functions were highly enriched in cluster 2 as compared with cluster 1. WGCNA identified 6 hub genes (*ACSL5, ALOX5AP, CD1D, CD74, IL4I1, and TBXAS1*) as FAM biomarkers. CM patients with high expression levels of the six biomarkers had better OS, PFS, and DSS than those with low expression levels of the biomarkers. The Cox regression analyses verified that the 6 FAM biomarkers can be independent prognostic factors for CM patients. The single-gene GSEA showed that the high expression levels of the 6 genes were mainly enriched in T-cell antigen presentation, the PD-1 signaling pathway, and tumor escape. The TME analysis confirmed that the FAM subtype biomarkers were not only related to immune infiltration but also highly correlated with immune checkpoints such as PD-1, PD-L1, and CTLA-4. TIDE scores confirmed that patients with high expression levels of the 6 biomarkers had worse immunotherapy responses. The 6 genes conveyed significant sensitivity to some chemotherapy drugs. qRT-PCR and IHC analyses verified the expression levels of the 6 biomarkers in CM cells.

**Conclusion:**

Our FAM subtypes verify that different FAM reprogramming affects the function and phenotype of infiltrating immune cells in the CM TME. The FAM molecular subtype biomarkers can be independent predictors of prognosis and immunotherapy response in CM patients.

## Introduction

Cutaneous melanoma (CM), the most fatal skin cancer, accounts for less than 5% of skin cancers but greater than 80% of skin cancer-caused deaths (1). Immunotherapy such as immune checkpoint inhibition (ICI) can significantly improve the prognosis of CM patients. However, up to 70% of CM patients have either innate or acquired ICI resistance, leading to high rates of recurrence, metastasis, and mortality (2–4). To provide these patients with evidence-based treatment and improve their clinical outcomes, we must be able to reliably predict immunotherapy responses.

The current Tumor Node Metastasis (TNM) classification of CM reflects primary tumor size and thickness, lymph node invasion, and distant metastasis based on the American Joint Committee on Cancer (AJCC)/Union for International Cancer Control(UICC) (5, 6), but it cannot predict prognosis and immunotherapy response in CM patients (7). Recent studies have demonstrated that, compared with the AJCC/UICC TNM classification system, classification systems that account for the immune component of the tumor microenvironment (TME) have superior prognostic value for predicting immunotherapy response (8, 9). For example, “hot” CM may respond well to immunotherapy, whereas “cold” CM with programmed cell death protein 1 (PD-1) inhibitor resistance, interferon γ inactivation, and CD8^+^ T-cell exhaustion may respond poorly to immunotherapy (10). Therefore, it is urgent to develop a CM classification system with promising biomarkers that predict the prognosis and immunotherapy response.

Abnormal metabolism promotes tumorigenesis and disease progression by disturbing the energy supply and regulating the TME (11, 12). Some metabolites, such as lactic acid, can inhibit the cytolytic ability of CD8^+^ effector T cells and downregulate immunity components in the TME (13). Tumor metabolic reprogramming plays a critical role in TME regulation and immunotherapy resistance (14–16). Metabolomic analyses have uncovered many novel biomarkers related to the diagnosis, prognosis, and treatment of many cancers, leading to the development of several antitumor strategies (17). As one of the most important intermediate products of lipid metabolism, fatty acid metabolism (FAM) is essential for many biological activities and maybe a promising immunotherapy target (18, 19). For example, Shang et al. (20) found that certain molecules can promote cervical cancer metastasis by reprogramming FAM. Ding et al. constructed a FAM signature that identified molecular subtypes of colorectal cancer and predicted prognosis and immunotherapy response (21). Zhang et al. reported that CD8^+^ tumor-infiltrating lymphocytes enhance the catabolism of fatty acids to preserve their effector functions and slow lung cancer progression (22). However, the prognostic and therapeutic value of FAM-related biomarkers in CM has not been reported. How CM cells sustain FAM in a metabolically dynamic TME and how FAM affects the phenotype and function of immune cells remain unclear.

The four aims of the present study were to 1) identify a FAM-related molecular subtype that represents the clinicopathological and immune features of CM; 2) identify and validate FAM-related genes (FAMGs); 3) unveil the association between FAM biomarkers and the immune phenotype and function of CM, and 4) determine the extent to which FAM molecular subtypes and biomarkers predict immunotherapy response.

## Materials and methods

### Collection of publicly available data

**Figure 1:**
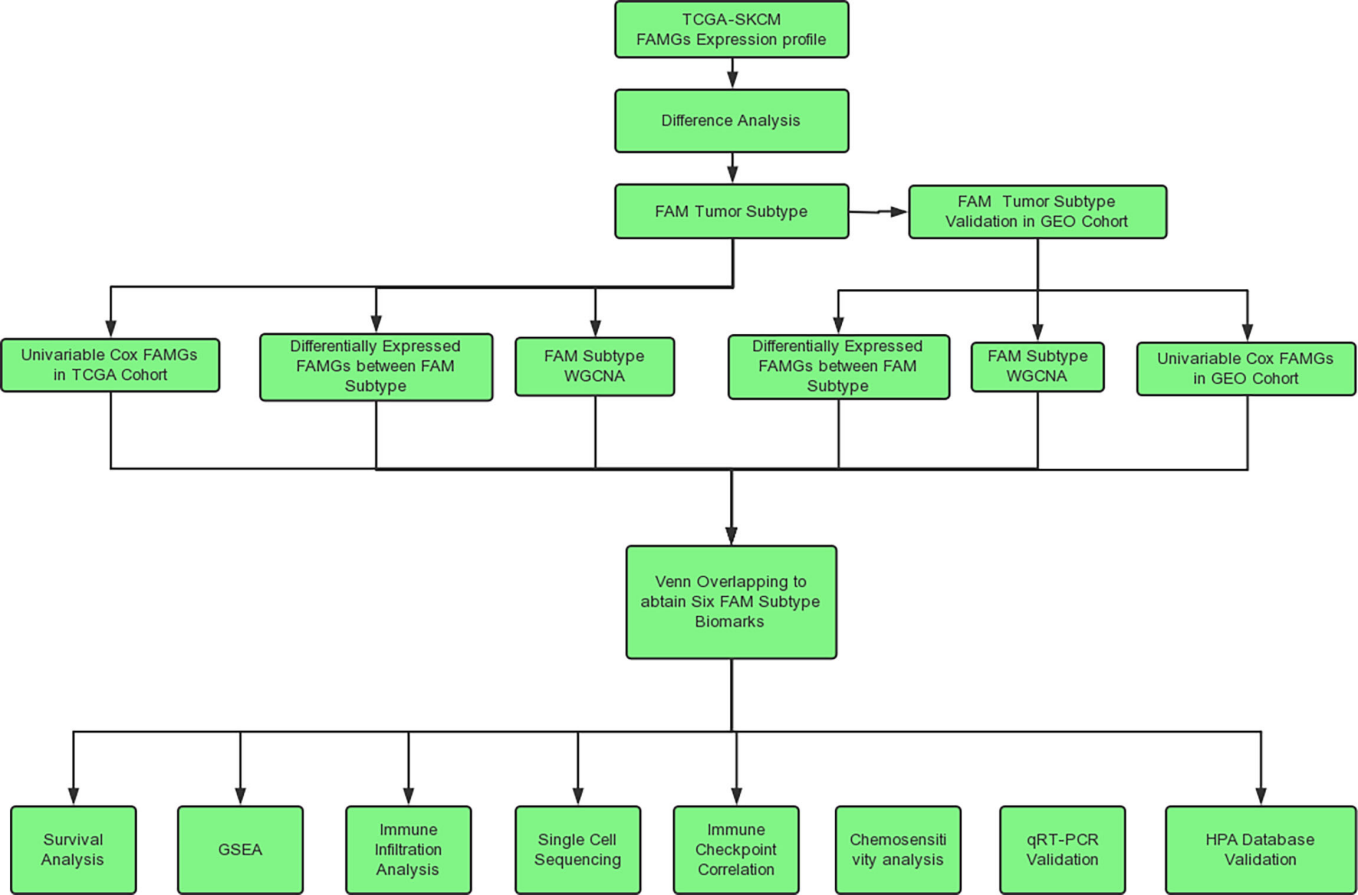
Flow chart.

The analysis process of this study is shown in **Figure 1**. The RNA sequencing data and corresponding clinical data of CM samples were downloaded from The Cancer Genome Atlas (TCGA) (http://cancergenome.nih.gov/) (23). The RNA sequencing data of normal skin samples were downloaded from the Genotype-Tissue Expression (GTEx) database (https://gtexportal.org/home/) (24). The expression values of all genes were publicly available and in level 3 HTseq fragments per kilobase of exon per million mapped fragments format. The merged RNA expression profile of the TCGA CM cohort (471 samples) and GTEx normal skin cohort (234 samples) was normalized, and batch effects between the TCGA and GTEX data were removed using the limma package in R (25). GSE65904 (26) and GSE72056 (27) were downloaded from the Gene Expression Omnibus (GEO) database (https://www.ncbi.nlm.nih.gov/) and used for molecular subtyping and single-cell validation. A total of 531 FAMGs were derived from 6 FAMG sets in the Molecular Signatures Database (http://www.gsea-msigdb.org/gsea/msigdb/) (28) and were listed in **Supplementary Table 1**. And the clinical detail of TCGA samples were listed in **Supplementary Table 2**

### Identification of differentially expressed FAMGs

To identify differentially expressed FAMGs (DEFAMGs), we analyzed the gene transcription data of the TCGA and GTEx cohorts using the limma package in R with a false discovery rate (FDR) < 0.05 and log2 fold change > 1. We used the pheatmap package in R to construct heatmaps of DEFAMGs. We used Gene Ontology (GO) (29) and Kyoto Encyclopedia of Genes and Genomes (KEGG) pathway (30) analyses to identify pathways in the Database for Annotation Visualization and Integrated Discovery associated with DEFAMGs (31). We used Metascape (https://metascape.org) (32) to verify and visualize the functional enrichment of the DEFAMGs.

### Identification and differential analysis of FAM molecular subtypes

We adopted non−negative matrix factorization (NMF) consensus clustering (33) to divide all TCGA CM patients into 2 molecular subtypes using the R “NMF” package based on FAMGs expression pattern. Principal component analysis (34) was used to visualize FAM molecular subtypes by dimensionality reduction. Overall survival (OS) and progression-free survival (PFS) were compared between the FAM molecular subtype groups. Gene set enrichment analysis (GSEA) (35) was performed to assess differences in immune-related pathways between the molecular subtypes. In addition, we used ESTIMATE (36) and ssGSEA (37) algorithms to compare the immune cell infiltration and function between the molecular subtypes. To validate the reliability of the FAM subtypes, we also performed the abovementioned analyses using a GEO dataset (GSE65904) as an external verification dataset.

We identified the DEFAMGs between FAM molecular subtypes in the TCGA and GEO datasets and performed GO and KEGG analyses. The protein-protein interaction (PPI) network was built on the intersection of the TCGA and GEO cohorts using STRING (https://cn.string-db.org/) (38) and reconstructed in Cytoscape v3.6 (39) to select hub biomarkers.

### Weighted gene co-expression network analysis

The expression profiles of the FAMGs were analyzed using a weighted gene co-expression network analysis (WGCNA) network (40) to select gene modules that were highly associated with FAM molecular subtypes. Among the soft threshold values, the β with the highest mean connectivity (β = 3) was chosen. The module eigengene was associated with molecular subtypes. Modules with the highest correlation were selected, and the genes in these modules were identified as genes related to FAM molecular subtypes. The minimum module size was 25. The module with the threshold of membership>0.4 and P value <0.05 was identified.

### Identification of hub biomarkers of FAM molecular subtypes

To identify hub biomarkers of FAM molecular subtypes, we used a Venn plot to identify overlapping PPI genes, WGCNA module genes, and DEFAMGs with statistical significance in univariable Cox regressions in both the TCGA and GEO datasets. We used correlation-based network analysis to identify interactions among core biomarkers. We analyzed the prognostic value of the hub biomarkers in predicting OS, PFS, and disease-specific survival (DSS) in the TCGA and GEO cohorts.

### GSEA of FAM biomarkers

To assess the immune-related and immune checkpoint–related pathways of the FAM biomarkers, we divided the patients in the TCGA CM cohort into groups with either high or low expression of each biomarker. The median expression level was used as the cut-off value. GSEA (41) was performed to identify significantly regulated pathways between the 2 groups. GSEA software was downloaded from the Broad Institute (http://www.broadinstitute.org/gsea/index.jsp). The gene sets of “c2.cp.kegg. v6.2. symbols”,”BIOCARTA_TCR_PATHWAY”,”REACTOME_PD_1_SIGNALING”,”LIN_TUMOR_ESCAPE_FROM_IMMUNE_ATTACK”,”WP_CANCER_IMMUNOTHERAPY_BY_PD1_BLOCKADE” and “WP_FERROPTOSIS” were downloaded from the Molecular Signatures Database (MSigDB). The normalized enrichment score (NES) was calculated for each gene set. Statistical significance was set at |NES| > 1, nominal P value < 0.05, and FDR q-value < 0.25.

### TME analysis

We used the ESTIMATE algorithm to calculate the immune score, tumor purity, ESTIMATE score, and stromal score for each CM sample. For each core biomarker, the differences between the high- and low-expression groups were analyzed using violin plots. Furthermore, the association between the expression of FAM biomarkers and immune cell infiltration was assessed by Pearson correlation analysis. The Tumor Immune Estimation Resource (TIMER) database (https://cistrome.shinyapps.io/timer/) (42) was used to identify correlations between immune cells and FAM biomarkers.

### Single-cell sequencing analysis

The GSE72056 single-cell dataset (4,645 single-cell sequencing samples of *Homo sapiens*; platform: GPL18573 Illumina NextSeq 500) was used to verify the expression of the FAMGs in the TME and assess the relationship between immune cells and FAMGs. We used the Seurat package in R for batch calibration and data normalization (43). We used the t-SNE package (44) to perform cell cluster analysis and used the SingleR package (45) to identify cell subpopulation annotations. We verified the connections between immune cells and molecular subtype biomarkers by evaluating the biomarker expression level of each cell.

### Correlation with immune checkpoints

We assessed differences in the expression of 30 genes that previous studies suggested to be immune checkpoint–related genes (46–48) between groups with high or low expression of each FAM biomarker. In addition, we used Pearson correlation analysis based on the TIMER database to assess associations between the expression of FAM biomarkers and that of key immune checkpoints, including PD-1, programmed death-ligand 1 (PD-L1), and cytotoxic T-lymphocyte antigen 4 (CTLA-4). We used TIDE scores to assess differences in immunotherapy response between groups with high or low expression levels of each FAM biomarker.

### Chemotherapy sensitivity analysis

We used the CellMiner database (https://discover.nci.nih.gov/cellminer) to construct an interaction network between chemotherapy sensitivity and FAM molecular subtype biomarkers. We performed Pearson correlation analysis to assess drug-gene associations.

### Human protein atlas database

The protein expression of the FAM biomarkers in normal and CM tissues was verified by analyzing immunohistochemical and immunofluorescent data extracted from The Human Protein Atlas (HPA; https://www.proteinatlas.org/).

### Cell culture and qRT-PCR

Human melanoma A375 cell line was purchased from American Type Culture Collection (Manassas, VA); the M14, human immortalized keratinocyte (HaCaT), and normal human skin melanocyte (PIG1) cell lines with STR certification were purchased from Shanghai Guandao Biological Engineering Company (Shanghai, China); and the SK-MEL-28 cell line was purchased from the Chinese National Infrastructure of Cell Line Resource. All cells were cultured in Roswell Park Memorial Institute (RPMI) 1640 medium supplemented with 10% fetal bovine serum at 37°C in a 5% CO_2_ atmosphere. Real-time quantitative polymerase chain reaction (qRT-PCR) was performed to determine relative gene expression levels. Total RNA was extracted with TRIzol reagent 24 hours. The concentration of RNA was determined by ultraviolet spectrophotometry. RNAs were reverse-transcribed into complementary DNAs (50 ng/µl) using a commercial complementary DNA reverse transcription kit. PCR with SYBR Green Master Mix (TaKaRa Bio, Kusatsu, Japan) was used to evaluate mRNA expression levels. Glyceraldehyde-3-phosphate dehydrogenase was used as the internal reference. All primers were synthesized by Servicebio Technology Co. (Wuhan, China). The primer sequences are shown in **Supplementary Table 3**. The PCR program consisted of an initial denaturation at 95°C for 10 minutes followed by 45 cycles of 95°C for 10 seconds, 60°C for 30 seconds, and 72°C for 20 seconds. Target gene expression was calculated using the 2^−ΔΔCT^ method.

### Statistical analysis

The survival, ggplot2, corrplot, pheatmap, singleR, and limma packages were executed using R software version 4.1.1 (https://www.r-project.org). A *P* value <0.05 was defined as statistically significant. The unpaired Student t-test was used to analyzing normally distributed continuous variables. Univariable Cox regressions and WGCNA were performed to identify FAMGs with significant prognostic values. Kaplan–Meier survival analyses and log-rank tests were used to assess OS, PFS, and DSS.

## Results

### DEFAMGs and functional enrichment

**Figure 2:**
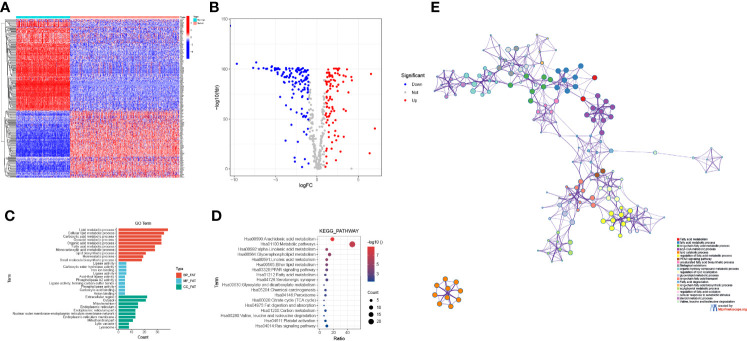
Identification of DEFAMGs in the TCGA cohort. **(A)** Heatmap of DEFAMGs. **(B)** Volcano plot of DEFAMGs. **(C)** GO enrichment analysis of DEFAMGs, showing only the first 10 terms respectively in the BP, MF and CC. **(D)** KEGG enrichment analysis of DEFAMGs, showing top 18 Fatty acid metabolism-related pathways. **(E)** Metascape displayed the functional enrichment network of fatty acid metabolism.

A total of 219 FAMGs were differentially expressed between CM tissues (n = 471) and normal skin tissues (n = 234), including 124 downregulated and 95 upregulated genes in the tumor samples (**Figures 2A, B**). GO analysis showed that the DEFAMGs were mainly enriched in lipid and FAM-related biological processes, cellular components, and molecular functions (**Figure 2C**). KEGG analysis showed that the DEFAMGs were mainly enriched in metabolic pathways and FAM pathways (arachidonic acid, linolenic acid, glycerophospholipid, etc.) (**Figure 2D**). Metascape functional annotation confirmed that DEFAMGs were mainly enriched in FAM processes (**Figure 2E**).

### FAM molecular subtypes

**Figure 3:**
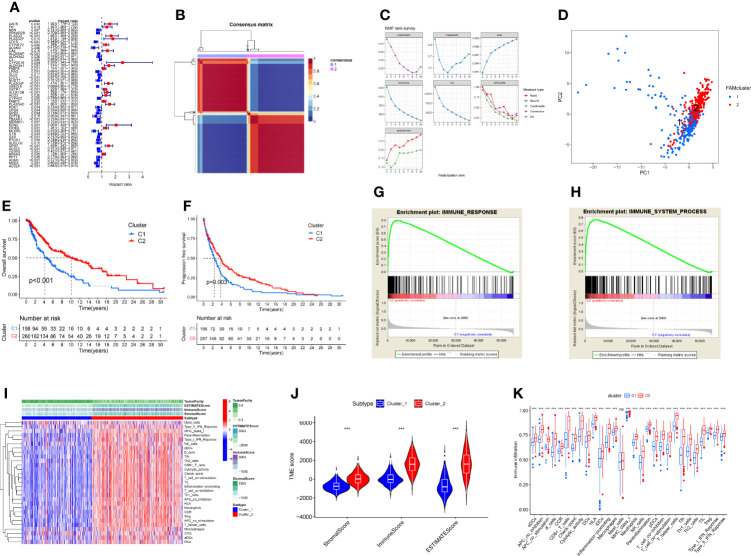
Construction of FAM molecular subtypes using TCGA CM samples. **(A)** A forest map of 49 prognostic FAMGs identified by univariable Cox regression. **(B)** The 2 FAM subtypes identified by the NMF algorithm. **(C)** The cophenetic, dispersion, and silhouette coefficients for the 2 FAM molecular subtypes. **(D)** Principal component analysis visualization of the 2 FAM molecular subtypes (cluster 1 and cluster 2). **(E, F)** The OS and DFS of patients with samples in cluster 2 were better than those of patients with samples in cluster 1. **(G, H)** GSEA results suggest that the immune system process and response pathways are upregulated in cluster 2. **(I)** Heatmap of TME components in the 2 FAM clusters, showing that cluster 2 had higher level of immune infiltrations than cluster 1. **(J)** Violin plot of TME ESTIMATE scores for the 2 FAM clusters. **(K)** Differences in immune infiltration and function between the 2 FAM clusters, showing immune functions were more activated in cluster 2 than cluster 1. ns, no sigificant, P>0.05; ***P≤0.001.

Univariable Cox regression revealed that 49 of the 219 DEFAMGs were significantly correlated with OS (**Figure 3A**). The NMF algorithm divided the TCGA CM samples into 2 clusters (cluster 1 and 2) (**Figure 3B**). The relationship between cophenetic, dispersion, and silhouette coefficients showed that the 2 clusters were significantly different (**Figure 3C**), which was further verified by principal component analysis (**Figure 3D**). Survival analysis showed that cluster 2 patients had better OS and DFS than cluster 1 patients did (**Figures 3E, F**). The GSEA results indicated that the immune response and immune system process were upregulated in cluster 2 (**Figures 3G, H**). Given the higher immune level and better prognosis in cluster 2, we compared the TME between the two clusters and found that immunocyte subpopulations were highly enriched in cluster 2 (**Figure 3I**). Compared with cluster 1, cluster 2 had significantly higher stromal, immune, and ESTIMATE scores (**Figure 3J**). The ssGSEA showed that cluster 2 also had higher levels of immune infiltration and more activated immune functions (**Figure 3K**). These findings were consistent with those of identical analyses performed using a GEO dataset (GSE65904) as an external validation dataset (**Supplementary Figures 1A–J**).

### DEFAMGs between FAM molecular subtypes

**Figure 4:**
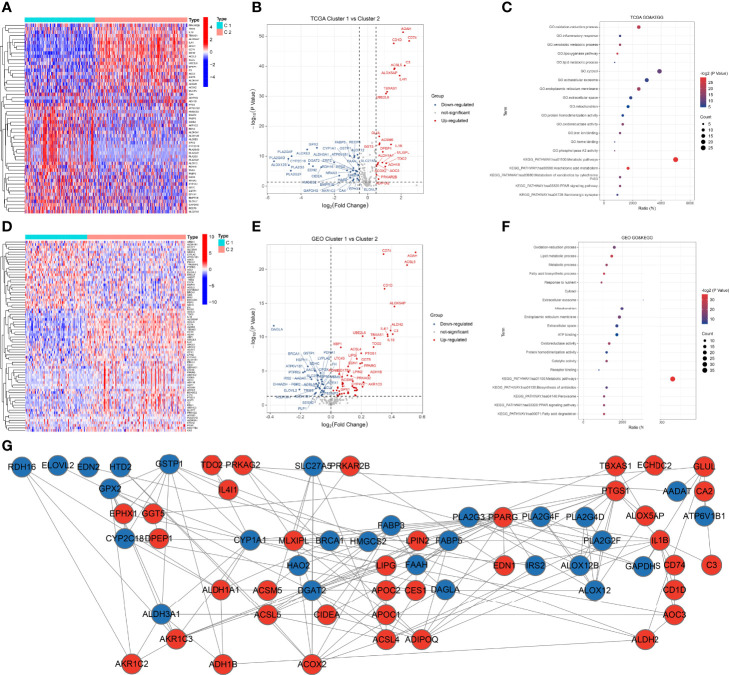
DEFAMGs between FAM molecular subtypes. **(A, B)** Heatmap and volcano plot of DEFAMGs between cluster 1 and cluster 2 in the TCGA cohort. **(C)** GO and KEGG enrichment analyses of DEFAMGs in the TCGA cohort. **(D, E)** Heatmap and volcano plot of DEFAMGs between cluster 1 and cluster 2 in the GEO cohort. **(F)** GO and KEGG enrichment analyses of DEFAMGs in GEO cohort. **(G)** The PPI network that includes an intersection of TCGA and GEO cohorts.

We identified 55 DEFAMGs between the 2 clusters of the TCGA CM samples (**Figure 4A**), including 22 upregulated and 33 downregulated genes in cluster 2 (**Figure 4B**). GO and KEGG analyses revealed that the DEFAMGs were enriched in the oxidation-reduction process, arachidonic acid metabolism, and metabolic pathways (**Figure 4C**). Using GEO samples, we identified 76 DEFAMGs between the 2 clusters (**Figure 4D**), including 48 upregulated and 28 downregulated genes in cluster 2 (**Figure 4E**). GO and KEGG analyses showed that these DEFAMGs were enriched in the oxidation-reduction process, the lipid metabolic process, and metabolic pathways (**Figure 4F**). The PPI network included 28 downregulated and 36 upregulated DEFAMGs (**Figure 4G**).

### WGCNA-selected FAM molecular subtypes modules and genes

**Figure 5:**
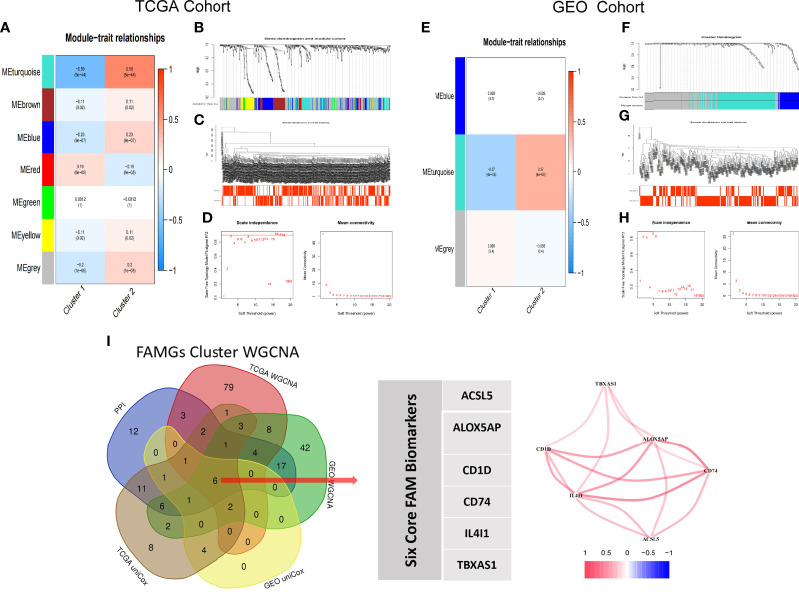
WGCNA-selected FAM molecular subtypes modules and genes. **(A–D)** TCGA Cohort WGCNA, **(A)** Seven modules are identified by WGCNA in TCGA samples; the turquoise module is highly correlation with FAM molecular subtypes (Cor= ± 0.59, p<0.001). **(B)** Dynamic Tree plot. **(C)** Sample heatmap of cluster 1 and cluster 2. **(D)** scale independence and mean connectivity. **(E–H)** Three modules are identified by WGCNA in GEO samples; the turquoise module is highly correlation with FAM molecular subtypes (Cor= ± 0.39, p<0.001). **(I)** Venn Diagram. ACSL5, ALOX5AP, CD1D, CD74, IL4I1 and TBXAS1 are identified as core biomarkers of FAM molecular subtype in CM patients.

We extracted the expressions of 531 FAMGs in TCGA samples and GSE65904 samples for WGCNA. Seven modules were identified by the average linkage hierarchical clustering based on the soft-thresholding power in TCGA samples, and the WGCNA traits heat map showed that the Module Eigengenes (ME) turquoise was selected (**Figure 5A**). The Module Eigengenes (ME) turquoise had the highest correlation with the FAM clusters (|cor| =0.59, P-value =5e-44) and contained 110 FAMGs. The dendrogram of genes clustered according to a dissimilarity measure (1-Topological Overlap Matrix, TOM) was shown in **Figure 5B**. The Sample dendrogram and soft threshold of WGCNA according to FAM Molecular subtypes were shown in **Figures 5C, D**. The co-expression network was constructed, and 3 modules were determined in GEO samples. Correlation analysis between the module eigengenes and FAM cluster showed that the ME turquoise (**Figures 5E–H**, Module–trait relationships = 0.39, P = 0.000) had the highest association with the FAM clusters. 92 genes in the module were considered to be hub FAM-related Modules genes. The overlapping part of the Venn plots identified 6 genes (i.e. ACSL5, ALOX5AP, CD1D, CD74, IL4I1 and TBXAS1) as core FAM biomarkers of CM (**Figure 5I**).

### Prognostic value of the 6 hub FAM biomarkers

**Figure 6:**
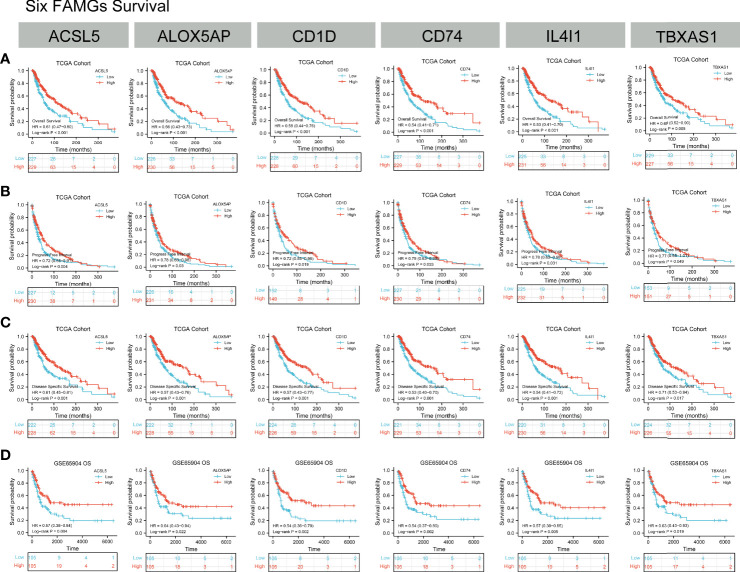
Kaplan-Meier analyses. **(A–C)** OS, PFS and DSS according to *ACSL5, ALOX5AP, CD1D, CD74, IL4I1*, and *TBXAS1* expression status in TCGA samples. **(D)** OS, PFS, and DSS according to *ACSL5, ALOX5AP, CD1D, CD74, IL4I1*, and *TBXAS1* expression status in GEO samples. K-M results showed the high-expression of *ACSL5, ALOX5AP, CD1D, CD74, IL4I1*, and *TBXAS1* had a better prognosis in CM patients.

Kaplan-Meier analyses showed that higher expression of *ACSL5, ALOX5AP, CD1D, CD74, IL4I1, or TBXAS1* was associated with better OS, PFS, and DSS in the TCGA cohort, and with better OS in GEO cohorts (**Figures 6A–D**). In addition, univariable and multivariable Cox regressions showed that the 6 genes were independent prognostic factors for OS (**Supplementary Table 4**).

### GSEA findings

**Figure 7:**
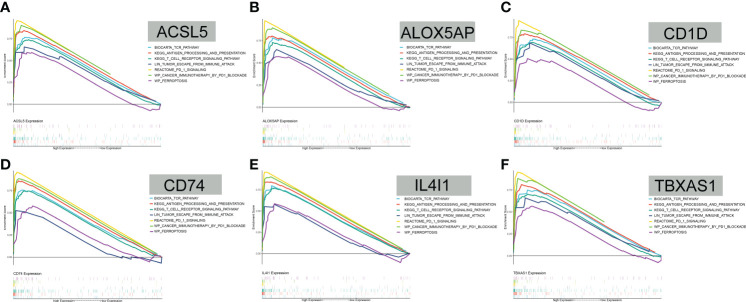
GSEA. **(A–F)** GSEA showed that antigen processing and presentation, T-cell receptor, the PD-1 signaling pathway, cancer immunotherapy by PD-1 blockade, tumor escape, and ferroptosis were all positively correlated with *ACSL5, ALOX5AP, CD1D, CD74, IL4I1*, and *TBXAS1* expression levels.

GSEA revealed that antigen processing and presentation, T-cell receptor, the PD-1 signaling pathway, cancer immunotherapy by PD-1 blockade, tumor escape, and ferroptosis were all positively correlated with the expression levels of the 6 FAMGs (**Figures 7A–F**). These results indicate that the 6 genes may be involved in tumor immune regulation, immune evasion, and tumor cell response to PD-1 blockade.

### TME analysis findings

**Figure 8:**
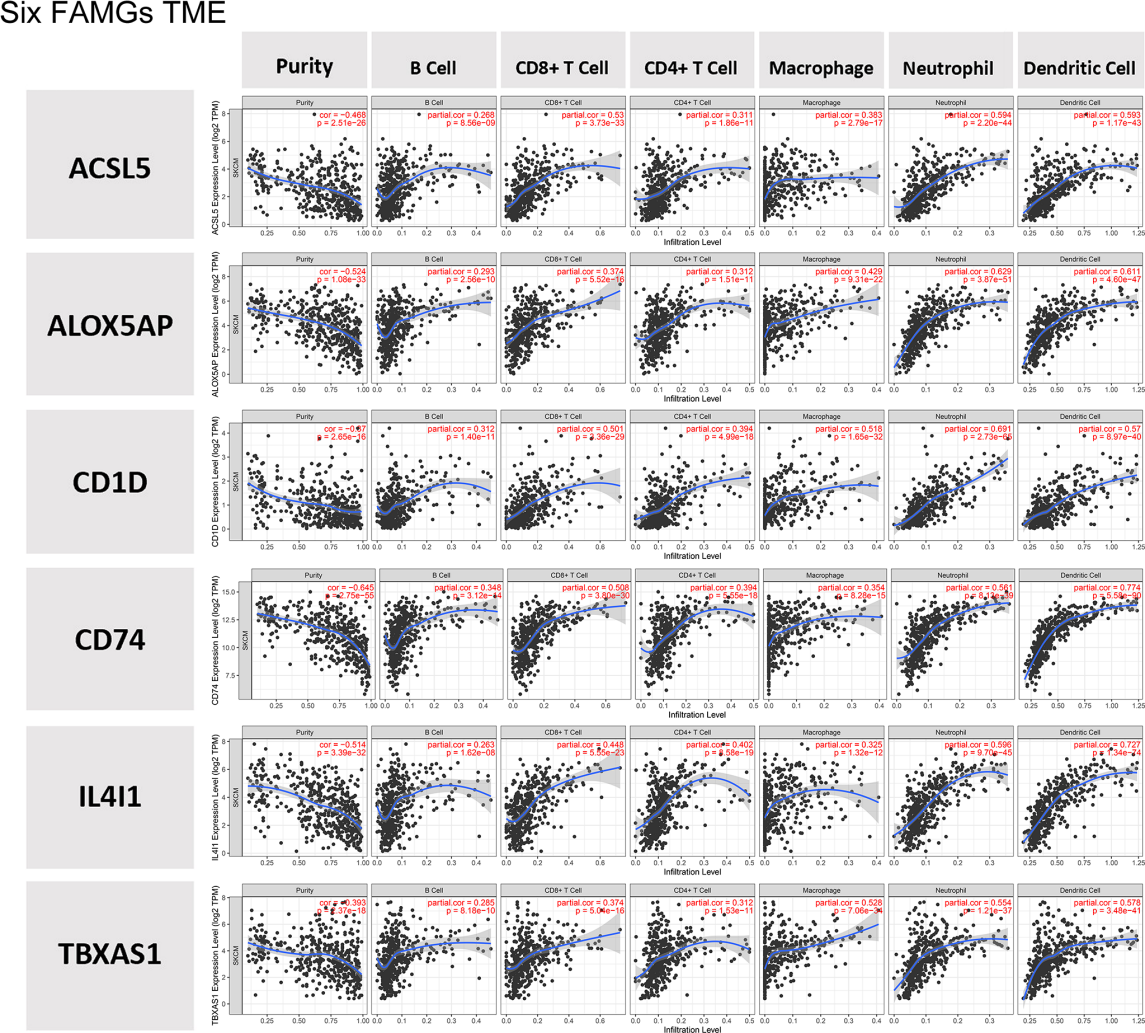
TME. The expression levels of the 6 FAMGs expression were positively correlated with the infiltration of B cells, CD8^+^ T cells, CD4^+^ T cells, macrophages, neutrophils, and dendritic cells.

Compared with the groups with low expression of the 6 FAMGs, those with high expression of the 6 FAMGs had significantly higher ESTIMATE, immune, and stromal scores but lower tumor purity (**Supplementary Figure 2**). The expression levels of the 6 FAMGs were significantly positively correlated with the ESTIMATE, immune, and stromal scores but negatively correlated with tumor purity (**Supplementary Figure 3**). The expression levels of the 6 FAMGs were also positively correlated with the infiltration of B cells, CD8^+^ T cells, CD4^+^ T cells, macrophages, neutrophils, and dendritic cells (**Figure 8**).

### Single-cell analysis findings

**Figure 9:**
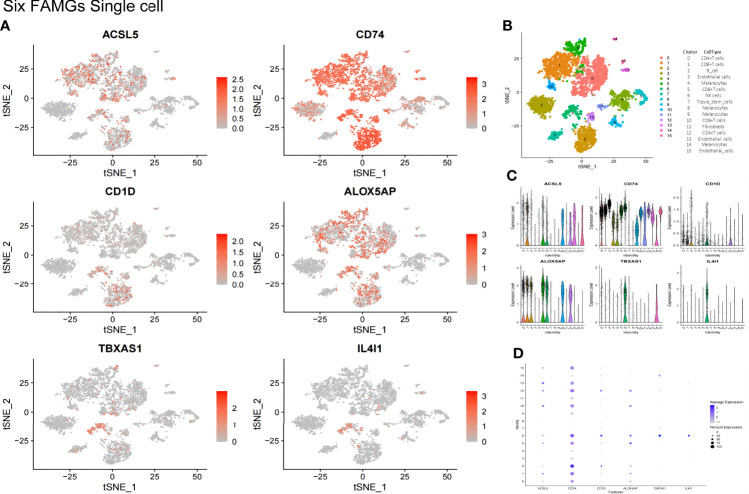
The expression of the 6 FAMGs was validated in a GEO single-cell cohort. **(A)** The expression levels of ACSL5, ALOX5AP, CD1D, CD74, IL4I1, and TBXAS1 were relatively high in the CD8^+^ T-cell, CD4^+^ T-cell, B-cell, and NK cell subsets. **(B)** Immunocyte annotation, Cluster 0, CD4+T cells; 1, CD8+T cells; 2, B cell; 3, Endothelial cells; 4, Melanocytes; 5, CD8+T cells; 6NK cells; 7, Tissue stem cells; 8, Melanocytes; 9, Melanocytes; 10, CD8+T cells; 11, Fibroblasts; 12, CD4+T cells; 13, Endothelial cells 14, Melanocytes;15, Endothelial cells. **(B)** A bubble chart of single-cell data shows the average and percent expression of *ACSL5, ALOX5AP, CD1D, CD74, IL4I1*, and *TBXAS1*. **(C)** A violin chart of single-cell data shows the expression levels of the 6 FAMGs. **(D)** A bubble chart of single-cell data shows the average and percent expression of *ACSL5, ALOX5AP, CD1D, CD74, IL4I1*, and *TBXAS1*.

The single-cell analysis confirmed that the expression levels of the 6 FAMGs were relatively high in the CD8^+^ T cell, B cell, and natural killer (NK) cell subsets (**Figures 9A, B**). The average and percent expressions of the 6 FAMGs are illustrated in a bubble chart of single-cell data (**Figure 9C**). The expression levels of the 6 FAMGs are illustrated in a violin chart of single-cell data (**Figure 9D**).

### Relationship between immune checkpoints and FAM molecular subtype biomarkers

**Figure 10:**
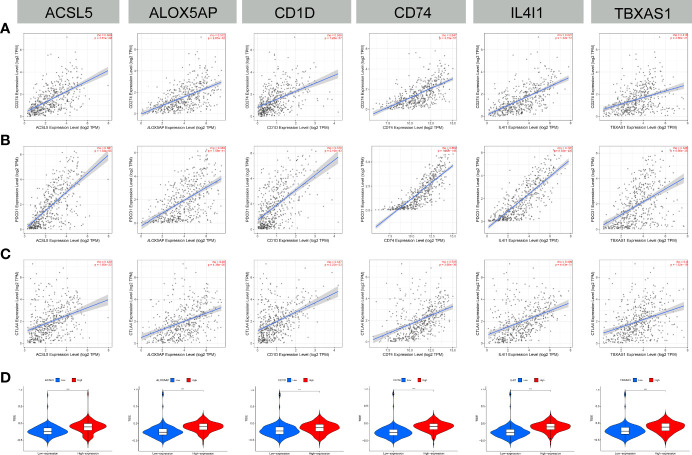
Immune checkpoint correlation and TIDE score. **(A–C)** The expression of the 6 FAMGs (ACSL5, ALOX5AP, CD1D, CD74, IL4I1 and TBXAS1) and that of immune checkpoints (PD-L1, PD-1, and CTLA-4) were significantly positively correlated. **(D)** The TIDE scores of the groups with high or low expression levels of the FAMGs showed that the high-expression group had worse immunotherapy responses. ***P≤0.001.

A total of 30 immune checkpoints, including PD-L1, PD-1, and CTLA-4, were found to be differentially expressed between groups with low or high expression of the 6 FAMGs (**Supplementary Figure 4**). The expression levels of the 6 FAMGs were significantly correlated with those of PD-L1, PD-1, and CTLA-4 (**Figures 10A–C**). A comparison of the groups’ TIDE scores demonstrated that the group with high expression of the 6 FAMGs had better immunotherapy responses (**Figure 10D**).

### Chemotherapy sensitivity

**Figure 11:**
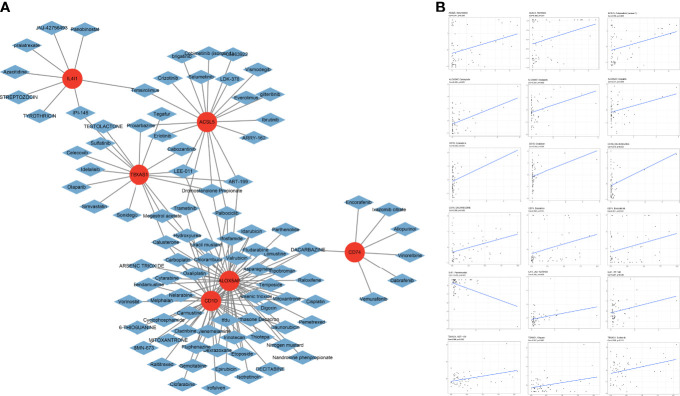
Chemotherapy sensitivity. **(A)** Chemotherapy sensitivity network shows that CM expressing the 6 genes has significant sensitivity to some chemotherapy drugs. **(B)** The scatter plot of the relationship shows that increased expression of *ACSL5, ALOX5AP, CD1D, CD74, IL4I1*, and TBXAS1 is associated with sensitivity to trametinib, selumetinib, cobimetinib, carboplatin, oxaliplatin, cisplatin, and dacarbazine.

Chemotherapy sensitivity analysis showed that positive expression of the 6 FAMGs was associated with high sensitivity to certain chemotherapy drugs (**Figure 11A**). Pearson correlation analysis confirmed that higher expression levels of the 6 FAMGs were associated with higher sensitivity to some chemotherapy and targeted drugs, such as trametinib, selumetinib, cobimetinib, carboplatin, oxaliplatin, cisplatin, and dacarbazine (**Figure 11B**).

### Validation of the 6 FAM molecular subtype biomarkers

**Figure 12:**
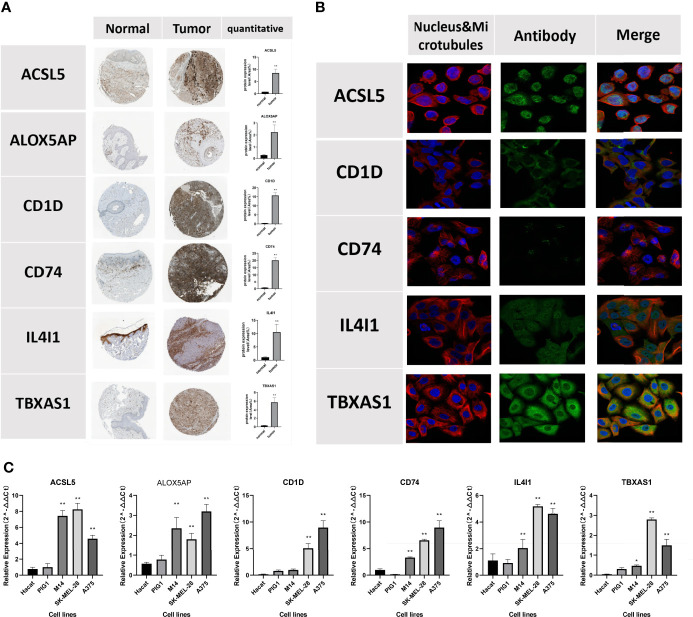
Verification of the protein expression of the 6 FAMGs using the HPA database. **(A)** Immunohistochemical images show the protein expression of ACSL5, ALOX5AP, CD1D, CD74, IL4I1, and TBXAS1 in CM and skin tissue. **(B)** Immunofluorescence show that *ACSL5, CD1D, CD74, IL4I1*, and *TBXAS1* are mainly located in the cytosol and nucleoplasm. **(C)** qRT-PCR, showing the relative expression of ACSL5, CD1D, CD74, IL4I1 and TBXAS1 were significant differences between 2 normal human cell lines and 3 melanoma cell lines. *P≥0.05; **P≥0.01.

Semiquantitative analyses of immunohistochemical images confirmed the expression levels of the 6 FAMGs were significantly higher in CM tissues than in normal skin (**Figure 12A**). Immunofluorescence analysis revealed that *ACSL5, CD1D, CD74, IL4I1, and TBXAS1* were mainly located in the cytosol and nucleoplasm (**Figure 12B**). qRT-PCR analysis revealed that the 6 FAMGs had significantly upregulated expression in A375, m14, and SK-MEL-28 cell lines compared with HaCaT and PIG1 cell lines (**Figure 12**).

## Discussion

### Major findings

This study comprehensively analyzed the role of FAMGs in the subtypes, prognosis, immune infiltration and immunotherapy response in CM. We first systematically investigated the correlation between DEFAMGs and the prognosis of patients with CM. Then we applied the NMF method to deconvolute the FAMGs expression profiles and identified 2 FAM clusters, cluster 1 and cluster 2, based on the TCGA CM cohort and further validated using the GEO cohort. According to the FAM subtypes, we identified 6 hub genes (*ACSL5, ALOX5AP, CD1D, CD74, IL4I1, TBXAS1*) as FAM subtype biomarkers. Finally, we verified the expression levels of the 6 genes in the HPA database and the qRT-PCR. Overall, our findings show that ACSL5, ALOX5AP, CD1D, CD74, IL4I1, and TBXAS1 are key FAM biomarkers that affect the function and phenotype of infiltrating immune cells and are associated with immunosuppression in the TME.

### FAM molecular subtype in CM

Tumor molecular subtypes that are based on predicted prognosis and TME composition have become a hotspot in cancer research, but few studies have provided comprehensive insight into the integral role of FAM in tumor molecular subtypes, especially in CM. For example, Ying et al. used a method of consensus clustering to build specific FAM-related molecular subtypes that were associated with malignancy and prognosis in glioma (49), but they did not explore the differences in immunocyte infiltration into the TME between the FAM-related molecular subtypes. Samson et al. constructed a mutational subtype of melanoma based on the BRAF mutation patterns of 2 FAM-related genes, *ALDH1A1* and *ALDH1A3*; however, this CM subtype accounts for ALDH1A3 expression as only a prognostic marker for BRAF/MEK inhibitor treatment response in BRAF-mutant metastatic melanoma patients (50). In the present study, we identified 2 FAM subtypes (cluster 1 and cluster 2) with different immune infiltration patterns based on the FAMG phenotype of CM. GSEA showed that immune response and immune system process were significantly regulated in FAM cluster 2. Immunocyte subsets, such as CD8^+^ T cells, B cells, tumor-infiltrating lymphocytes, T follicular helper cells, regulatory T cells, and T helper cells, were highly enriched in FAM cluster 2, indicating that cluster 2 had higher levels of T-cell subset infiltration than cluster 1 did.

T cells degrade fatty acids through fatty acid oxidation to acquire lipids for energy. In addition, the development and function of different T-cell subsets are closely linked to the balance between fatty acid synthesis and fatty acid oxidation in the TME. For example, the preferential usage of fatty acid oxidation has been linked to the development of CD8^+^ memory T cells and the induction of CD4^+^ regulatory T cells over other T-cell lineages. Especially in CD4^+^ T-cell subsets, the FAM profile changes during an immune response and is influenced by different tissues and different types of inflammation. Our FAM subtypes provide further evidence that different FAM patterns affect the abundance of T-cell subsets in the CM TME. Recent studies confirmed that T-cell subpopulations are the targets of ICI and chimeric antigen receptor T-cell therapy and that the status of T cells can strongly influence patients’ prognosis (51, 52). Our results also showed that FAM cluster 2, with its high levels of T-cell infiltration, was associated with favorable outcomes, which suggests that cluster 2 may be more responsive to immunotherapy than cluster 1. In addition, our GO and KEGG analyses showed that the DEFAMGs between cluster 2 and cluster 1 were mainly enriched in the oxidation-reduction process, peroxisome proliferator-activated receptor (PPAR) signaling pathway, and lipid metabolic pathways. Accumulating evidence shows that the PPAR signaling pathway, which is involved in lipid metabolism, energy homeostasis maintenance, inflammation, and immune tolerance, has a role in carcinogenesis. Lee et al. found that AMP-activated kinase induces the upregulation of CPT1 and PGC-1 to activate the PPAR signaling pathway, which may more indirectly favor fatty acid oxidation (53). Therefore, the PPAR signaling pathway may be a key determinant of FAM patterns between FAM subtypes, and it may be used in the context of novel therapeutic strategies against CM.

### FAM molecular subtype biomarkers

Based on the FAM molecular subtypes, we identified the 6 FAMGs (*ACSL5, ALOX5AP, CD1D, CD74, IL4I1, TBXAS1*) that play vital regulatory roles in the melanoma TME. Among the 6 hub FAMGs, IL4I1 (interleukin-4-induced gene 1) has a vital role in immunosuppressive functions and tumor immune escape. In 2009, Carbonnelle et al. were the first to propose that the novel immunosuppressive enzyme IL4I1, which is produced by the neoplastic cells of several B-cell lymphomas and by tumor-associated macrophages, is a prognostic biomarker and therapeutic target in cancer (54). Later, Fanette et al. found that tumor-associated macrophages with high IL4I1 expression can inhibit T-cell proliferation *in vitro* through H_2_O_2_ production. They also confirmed that in human melanoma and mesothelioma, minimal IL4I1 activity-induced tumor escape was preceded by a rapid diminution of interferon γ–producing cytotoxic antitumor CD8^+^ T cells (55). Cousin et al. further showed that IL4I1 stimulates the generation of Foxp3^+^ regulatory T cells and limits T helper 1 and T helper 2 polarization *in vitro*, and their findings reinforced the concept that IL4I1 facilitates tumor escape from the immune response (56). Sadik et al. recently identified IL4I1 as a major aryl hydrocarbon receptor (AHR)-activating enzyme that promotes AHR-driven cancer cell motility and suppresses adaptive immunity. Compared with IDO1 or TDO2, IL4I1 had a stronger effect on AHR activity, which suggests that IL4I1 is an alternative metabolic immune checkpoint that could be therapeutically targeted in patients in whom combined ICI and IDO1 inhibition therapy has failed (57).. In the present study, we consistently observed that the PD-1, T-cell receptor, and tumor escape from immune attack signaling pathways were significantly enriched in CM samples with high IL4I1 expression. The differential expression of IL4I1 was significantly correlated with the infiltration of immune cells such as B cells, CD8^+^ T cells, macrophages, and neutrophils in the TME. These findings suggest that IL4I1 can be an indicator of response to anti–PD-1 treatments and is a novel metabolic immune checkpoint in CM.

Previous studies showed that *ACSL5* (acyl-CoA synthetase 5), a nuclear-coded FAMG expressed in the mitochondria, can convert carbons from citrate to bioactive fatty acids to stimulate the inflammatory response in the TME (58, 59). For example, Klaus et al. found that the high activity of *ACSL5* enhances caspase-3 and caspase-7 activity to promote apoptosis regulated by TP53 status *via* WNT2B palmitoylation in enterocytes and colorectal adenocarcinomas (60). In addition, *ACSL5* can be used to predict survival and immunotherapy response. Gassler et al. showed that lower *ACSL5* expression is a prognostic marker for early recurrence in patients with colorectal adenocarcinoma (61). Chen et al. showed that breast, colorectal, lung, or ovarian cancer patients who have higher *ACSL5* expression have good survival outcomes (62). Our results showed that *ACSL5* is an important indicator of the OS, PFS, and DSS of CM patients. We also found that *ACSL5* is correlated with the ferroptosis signaling pathway in CM, which suggests that *ACSL5* can inhibit the proliferation of tumor cells by inducing ferroptosis.

*ALOX5AP* is a key enzyme that facilitates the activity of 5-lipoxygenase (5-LOX), which metabolizes arachidonic acid to leukotrienes. Moore et al. found that the 5-LOX/ALOX5AP pathway can affect cancer-related immune evasion in the TME (63). Ye et al. confirmed that *ALOX5AP* is involved in M2 macrophage recruitment, infiltration, and polarization, which can indirectly block tumor-specific T-cell activity to promote immune evasion in the ovarian cancer TME (64).

*CD1D*, a major histocompatibility complex class I–like molecule, presents lipoidal antigen to NK T cells, which are involved in the innate anticancer immune response. Bernal et al. confirmed that the CD1D molecule plays a crucial role in the induction of melanoma immune evasion by forming a complex with β2 microglobulin in the CD1D/NK T-cell axis (65). CD1D^+^ tumors can evade recognition by NK T cells in the CD1D/NK T-cell axis by shedding glycolipids, which presumably replace the endogenous lipids that are bound to *CD1D* and cannot be recognized by NK T cells (66).

*CD74* has been implicated to play a tumor-progression role in the immune microenvironment of CM patients. Figueiredo et al. confirmed that *CD74*, as a macrophage migration inhibitory factor (MIF), regulates the activity of macrophages and other immune cells *via* the CD74-MIF signaling pathway. Interfering with MIF-CD74 immunosuppressive signaling can restore the antitumor immune response in metastatic melanoma (67). According to other studies, the polarization of macrophages to M2 macrophages induces immunosuppression by suppressing cytotoxic T cells in the TME (68). In the present study, we also showed that *CD74* is positively associated with the infiltration of macrophages, dendritic cells, and neutrophils in CM. Our single-cell analysis of the TME further showed that high *CD74* expression was enriched in CD8^+^ T cells, CD4^+^ T cells, macrophages, neutrophils, and dendritic cells in the CM TME.

In the arachidonic acid cascade, *TBXAS1* encodes for thromboxane synthase, which converts prostaglandin H2 into thromboxane A2, a process that involves the modulation of cell cytotoxicity and tumor growth and metastasis (69, 70). Abraham et al. showed that *TBXAS1* genes are associated with breast cancer risk (71). We found that TBXAS1 is significantly correlated with neutrophils in the TME, indicating that high TBXAS1 expression upregulates the inflammation response in the TME.

### Limitations

Although it presents encouraging results, our study had several limitations. First, the FAM tumor subtype was constructed and validated using open data sources (TCGA and GEO). Testing the subtype in an independent patient cohort would validate the reliability of our subtypes in identifying CM patients. Second, our study lacked *in vivo* experiments to verify the molecular function of the 6 hub genes. Future studies are needed to investigate the mechanisms underlying the 6 FAM biomarkers’ mediation of CM progression and the immune microenvironment.

## Conclusion

Our FAM subtypes verify that different FAM reprogramming affects the function and phenotype of infiltrating immune cells in the CM TME. Our findings suggest that the FAM molecular subtype biomarkers *ACSL5, ALOX5AP, CD1D, CD74, IL4I1*, and *TBXAS1* can be independent predictors of prognosis and immunotherapy response in CM patients. These findings may provide potential therapeutic targets in CM.

## Data availability statement

The datasets presented in this study can be found in online repositories. The names of the repository/repositories and accession number(s) can be found in the article/**Supplementary Material**.

## Author contributions

YX and YBC performed the data procedures and the writing. WJ, XY, and DC were involved in the review of the manuscript. YC and JZ contributed to data management, QZ and YH provided the idea for this study. All authors contributed to the study conception and design and approved the final manuscript.

## Acknowledgments

We are grateful to those who provide and maintain the public databases and datasets we used in this study. We thank Joseph Munch in MD Anderson’s Research Medical Library for editing the manuscript.

## Conflict of interest

The authors declare that the research was conducted in the absence of any commercial or financial relationships that could be construed as a potential conflict of interest.

## Publisher’s note

All claims expressed in this article are solely those of the authors and do not necessarily represent those of their affiliated organizations, or those of the publisher, the editors and the reviewers. Any product that may be evaluated in this article, or claim that may be made by its manufacturer, is not guaranteed or endorsed by the publisher.
