# Supplementary figures and images for: Identification of fatty acid metabolism–related molecular subtype biomarkers and their correlation with immune checkpoints in cutaneous melanoma
Source: Front Immunol. 2022 Nov 18;13:967277. doi: 10.3389/fimmu.2022.967277 (PMC9716430; doi:10.3389/fimmu.2022.967277)

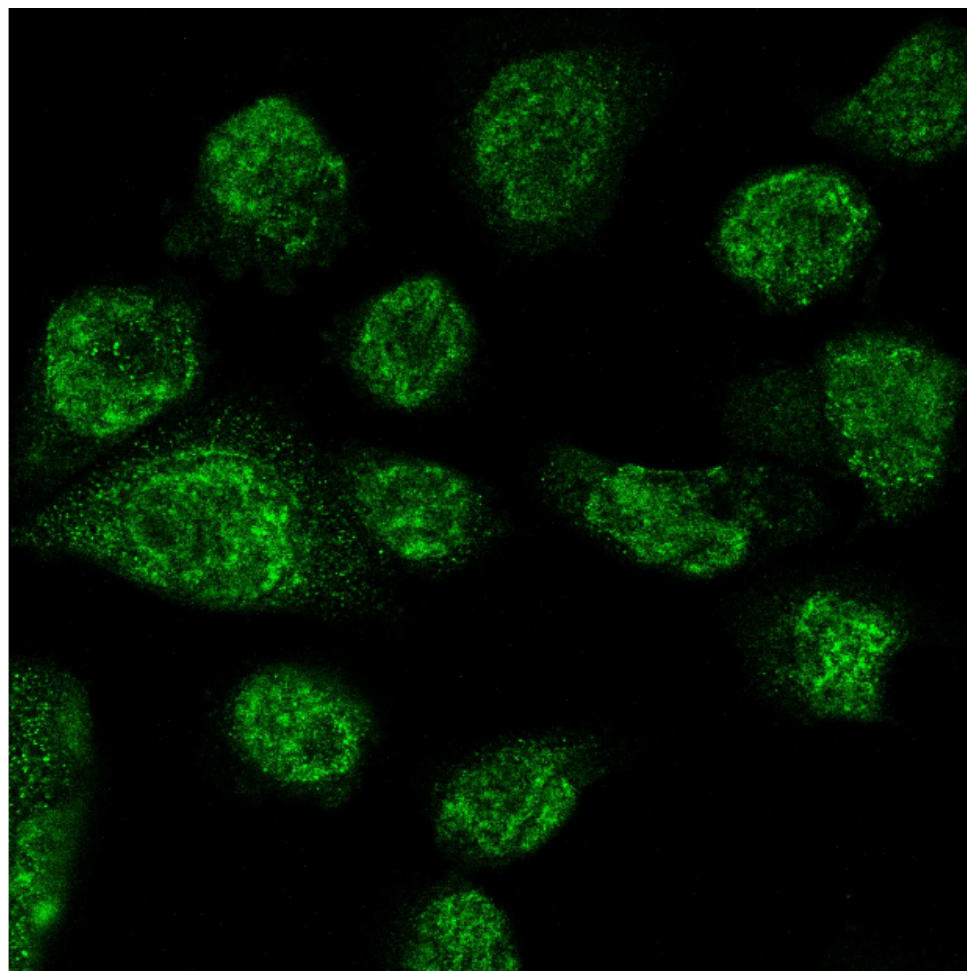

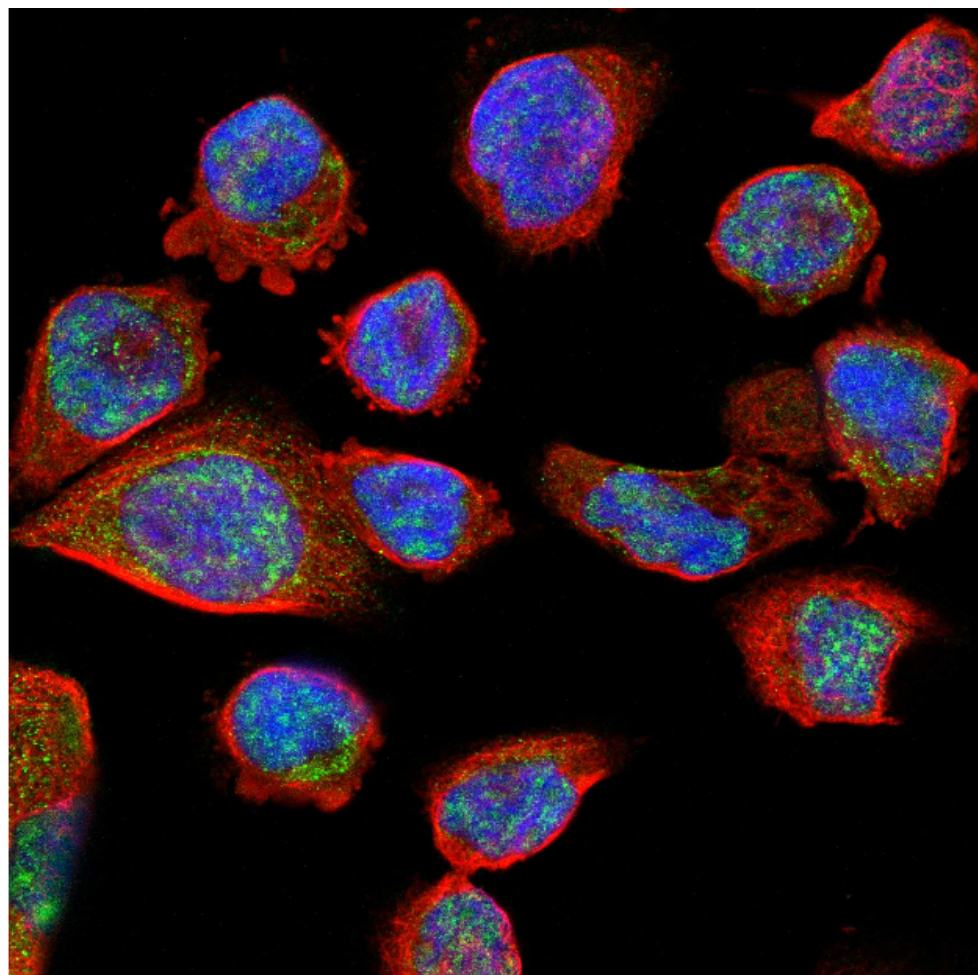

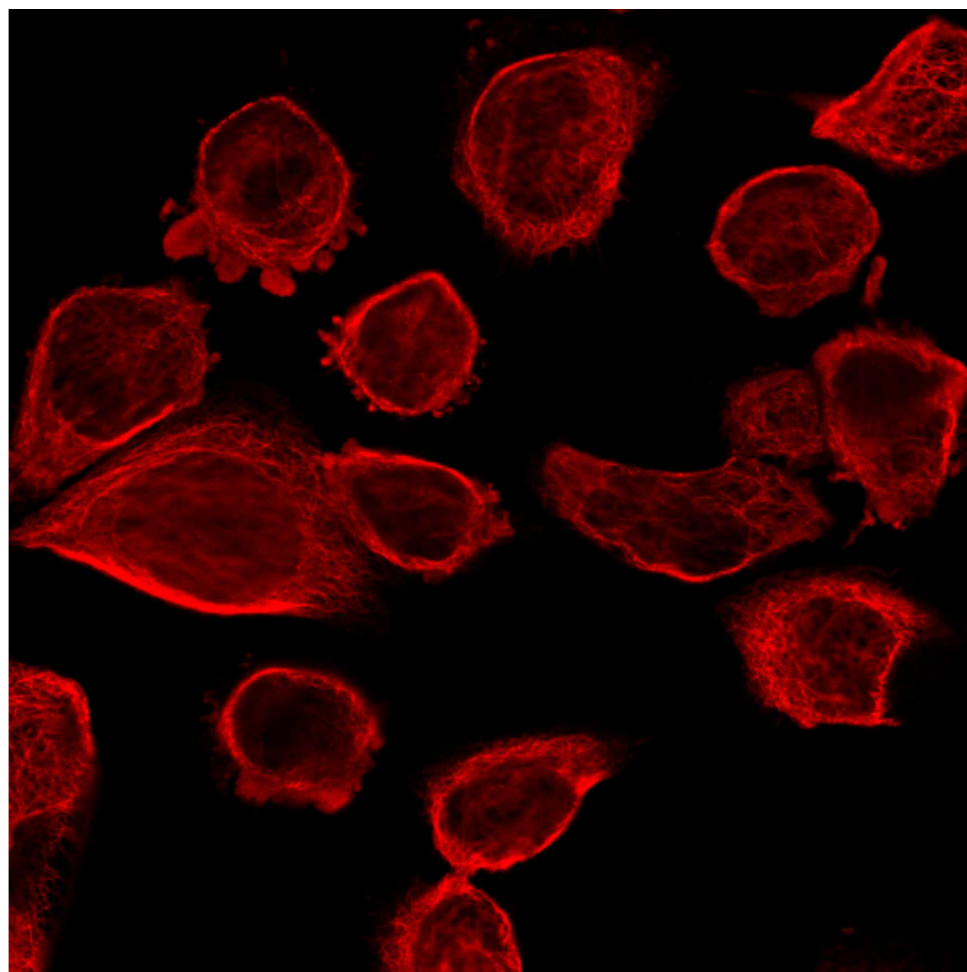

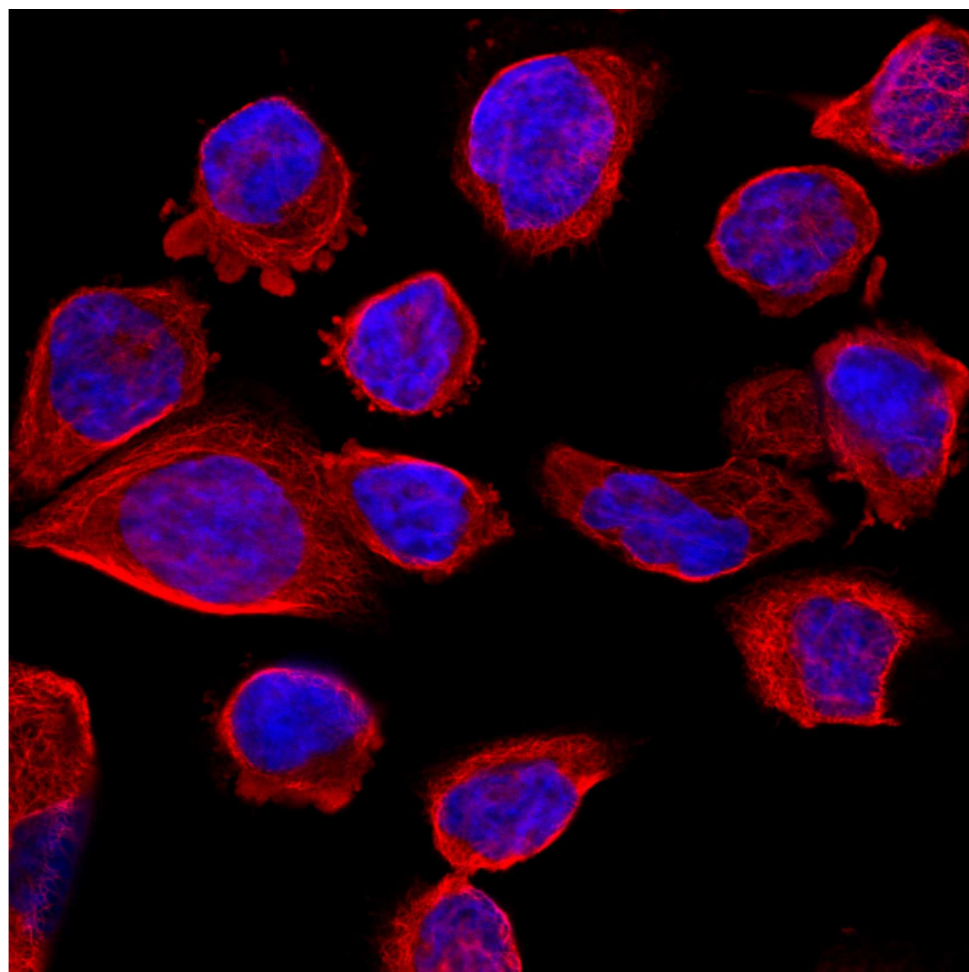

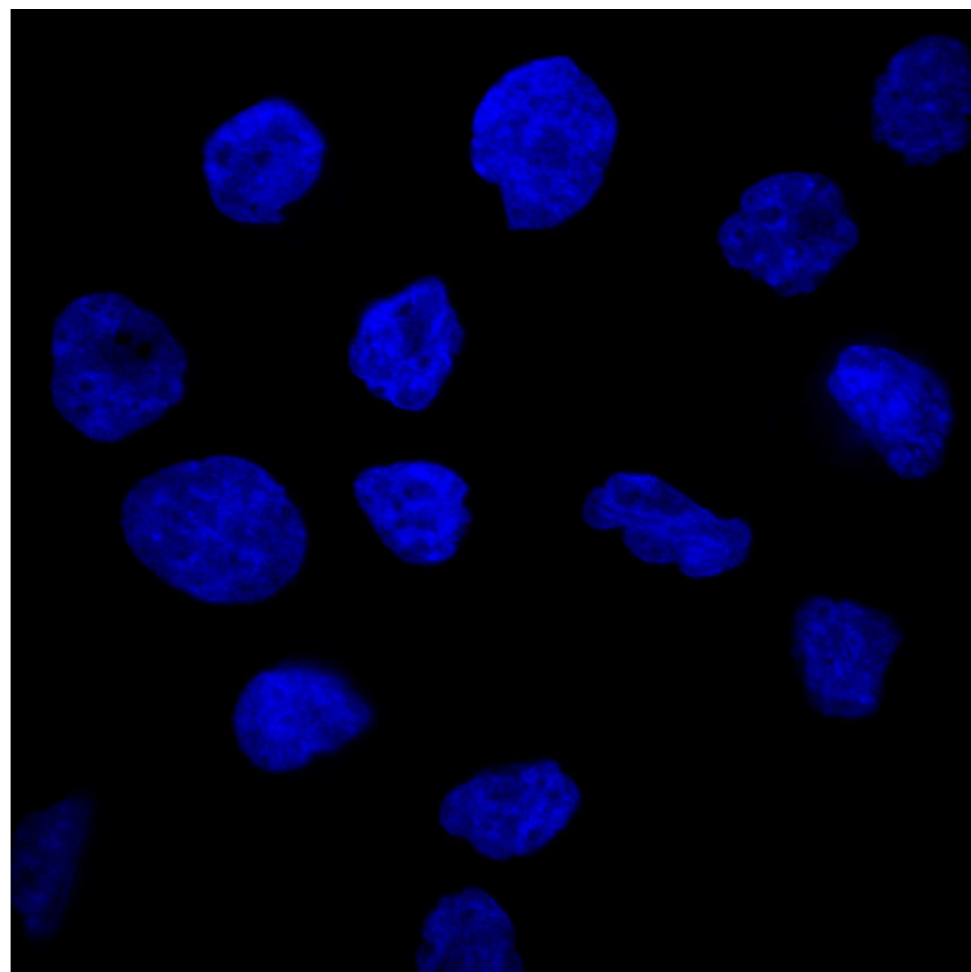

Supplement: Supplementary file 1 [file DataSheet_1.pdf]

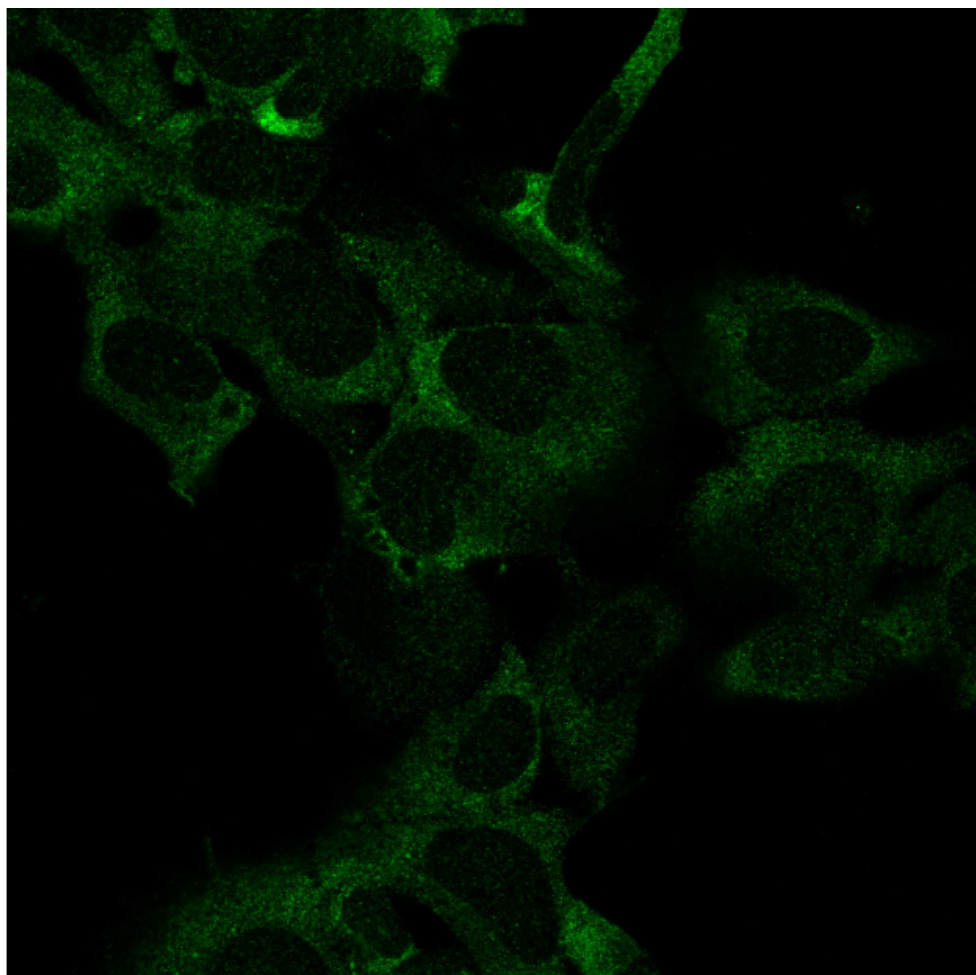

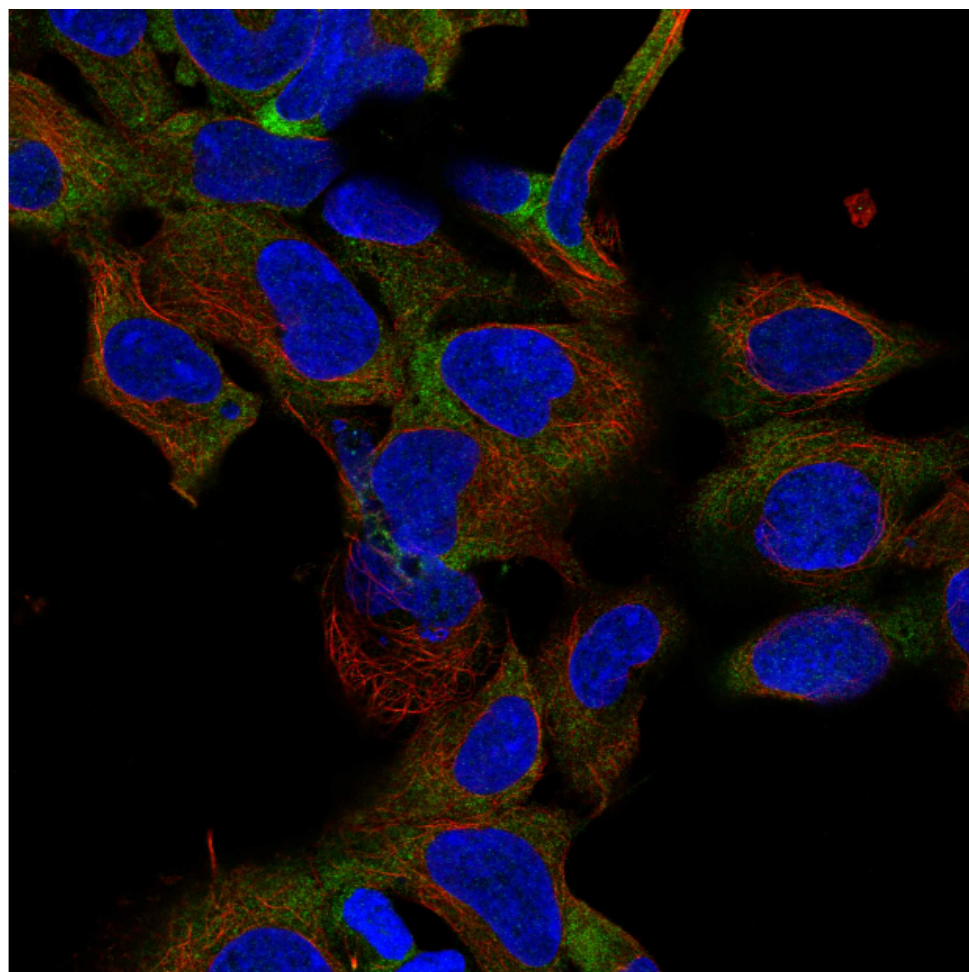

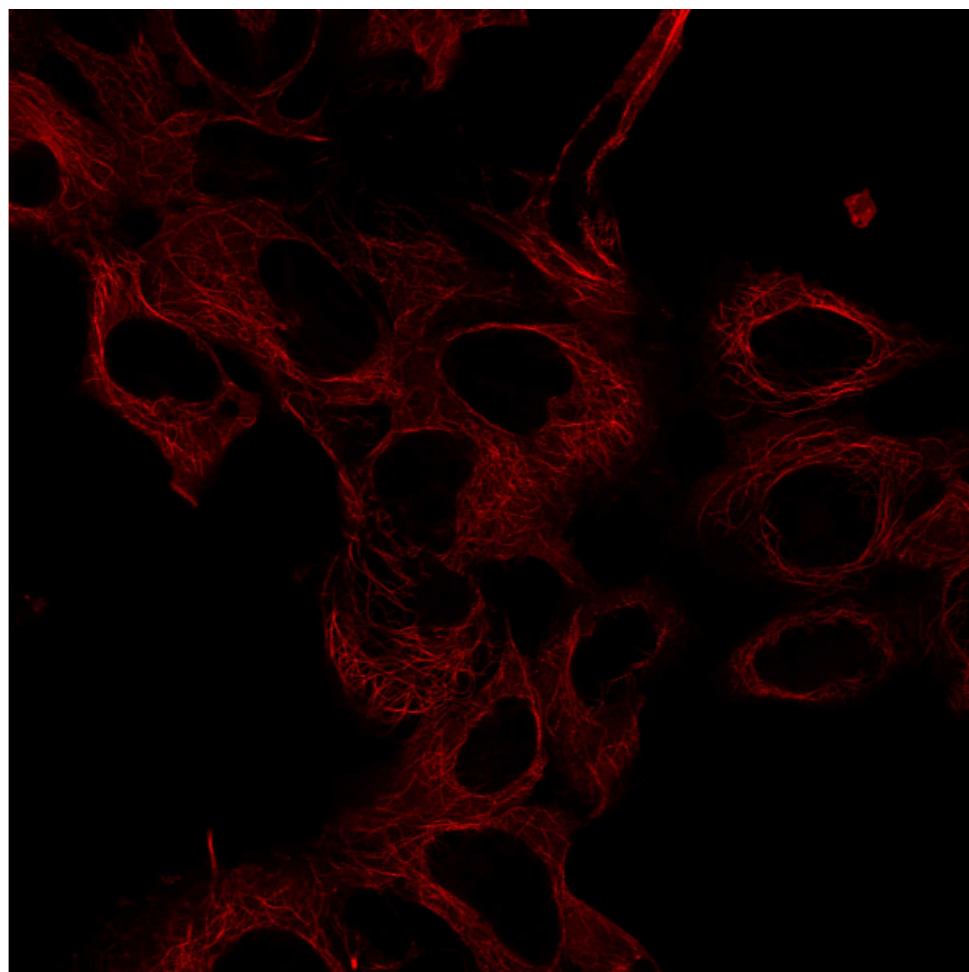

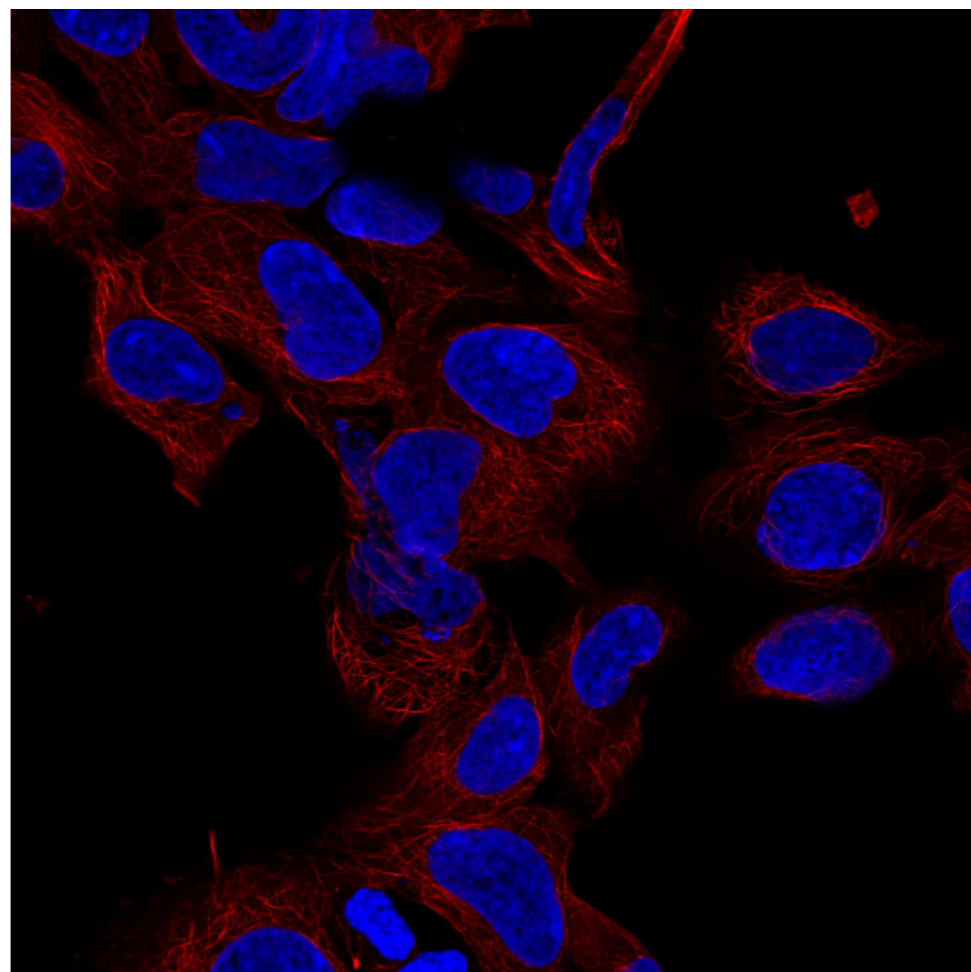

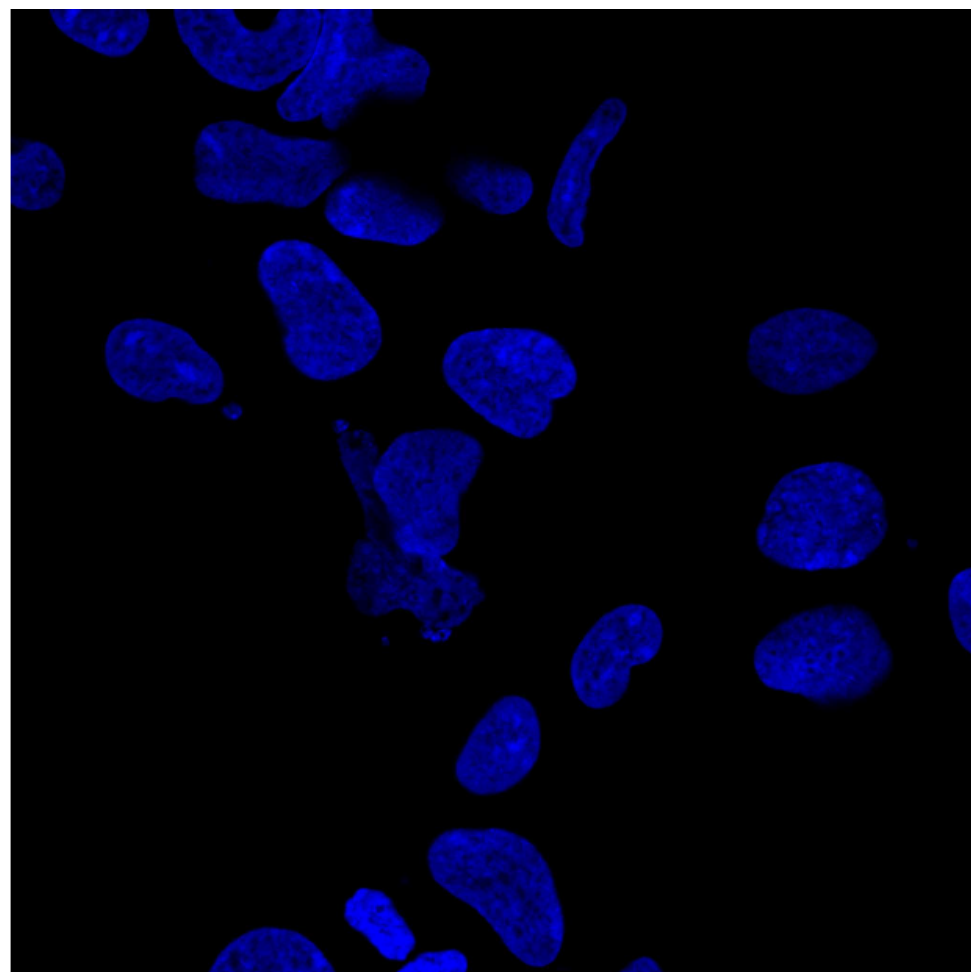

Supplement: Supplementary file 2 [file DataSheet_2.pdf]

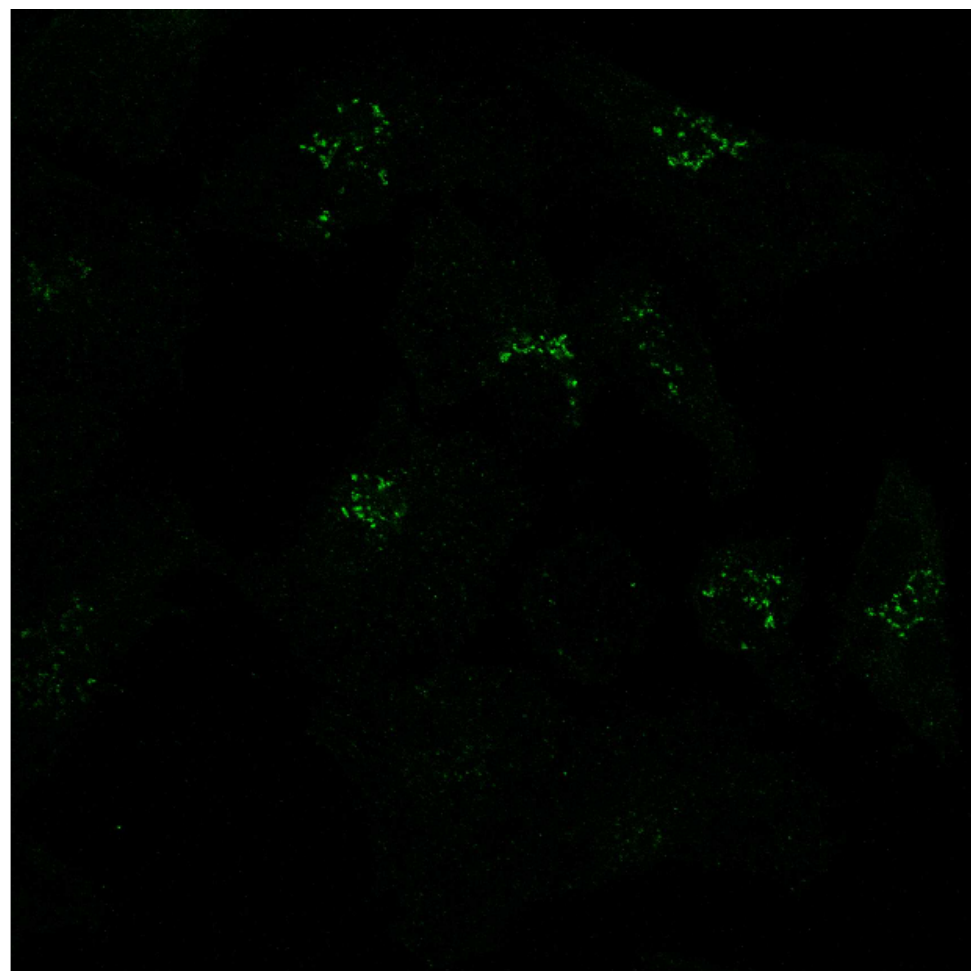

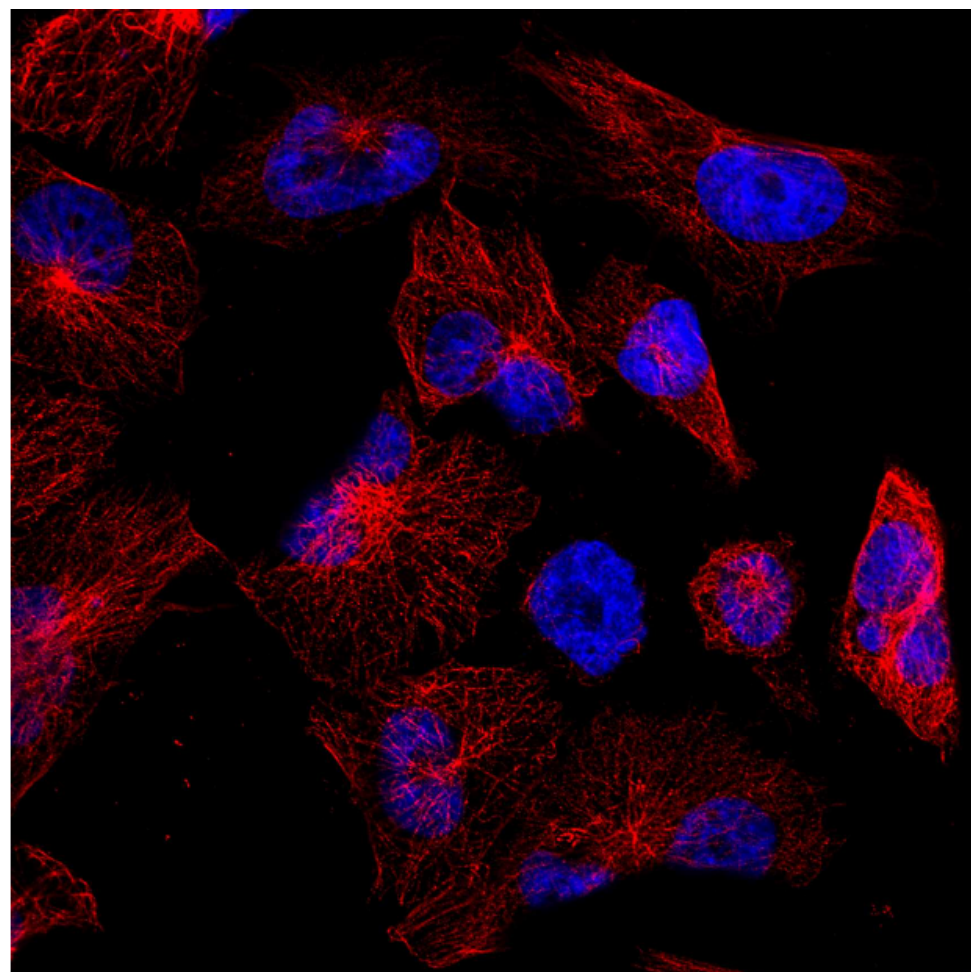

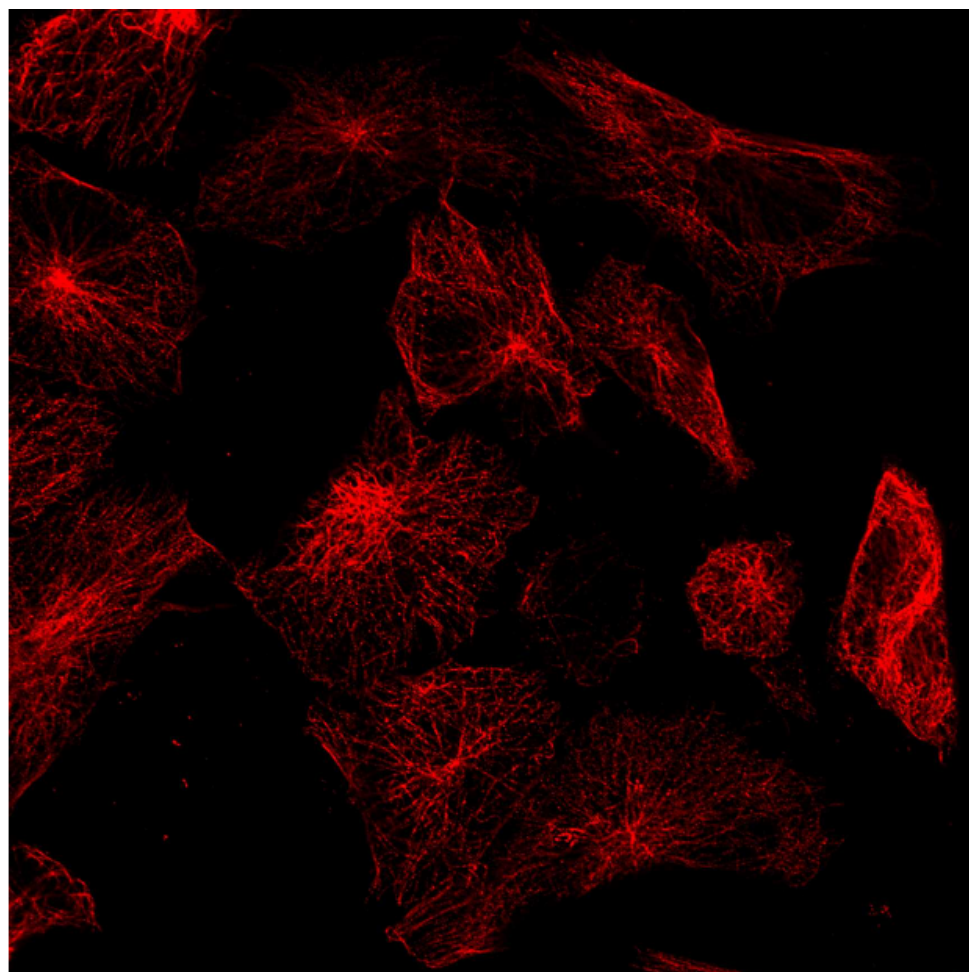

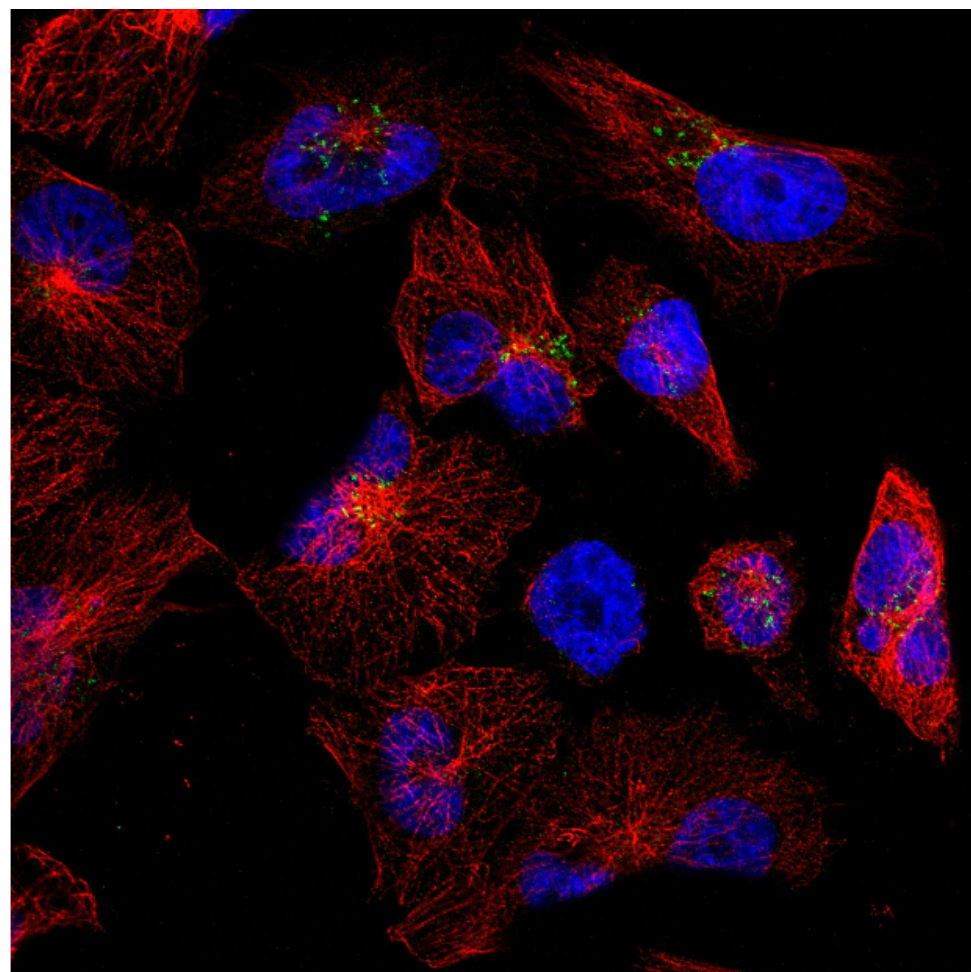

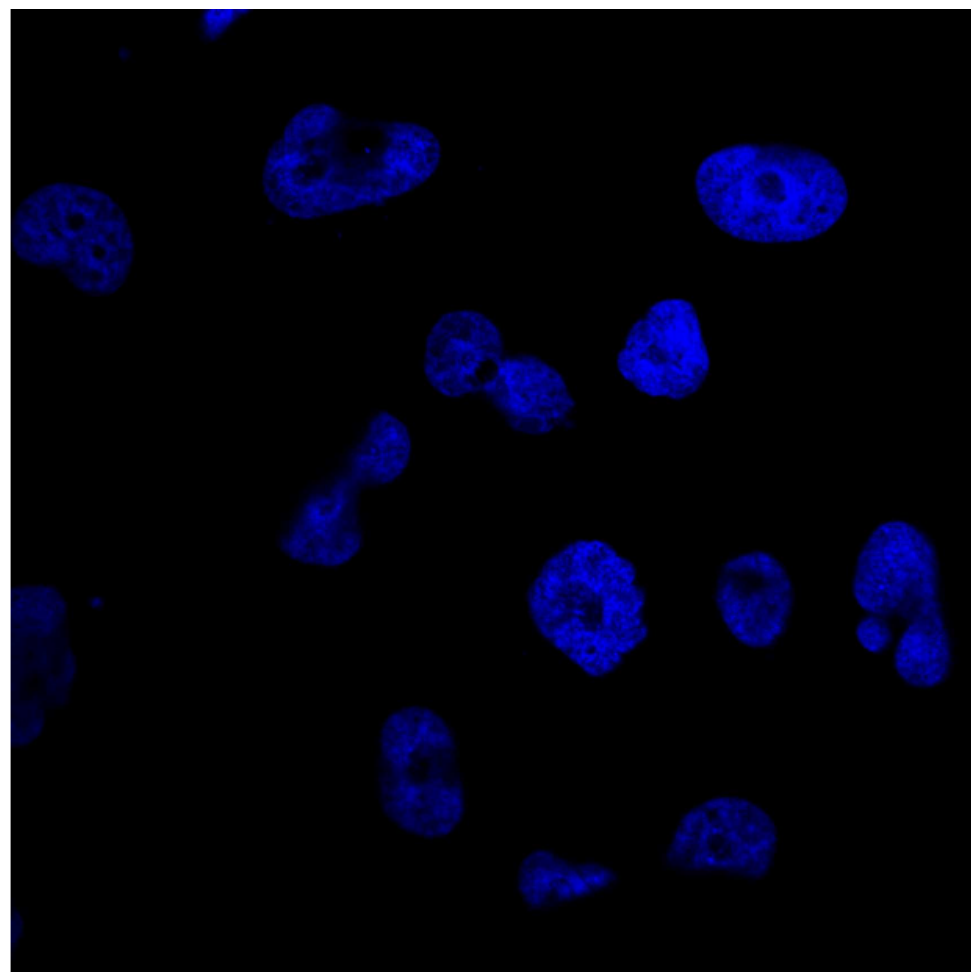

Supplement: Supplementary file 3 [file DataSheet_3.pdf]

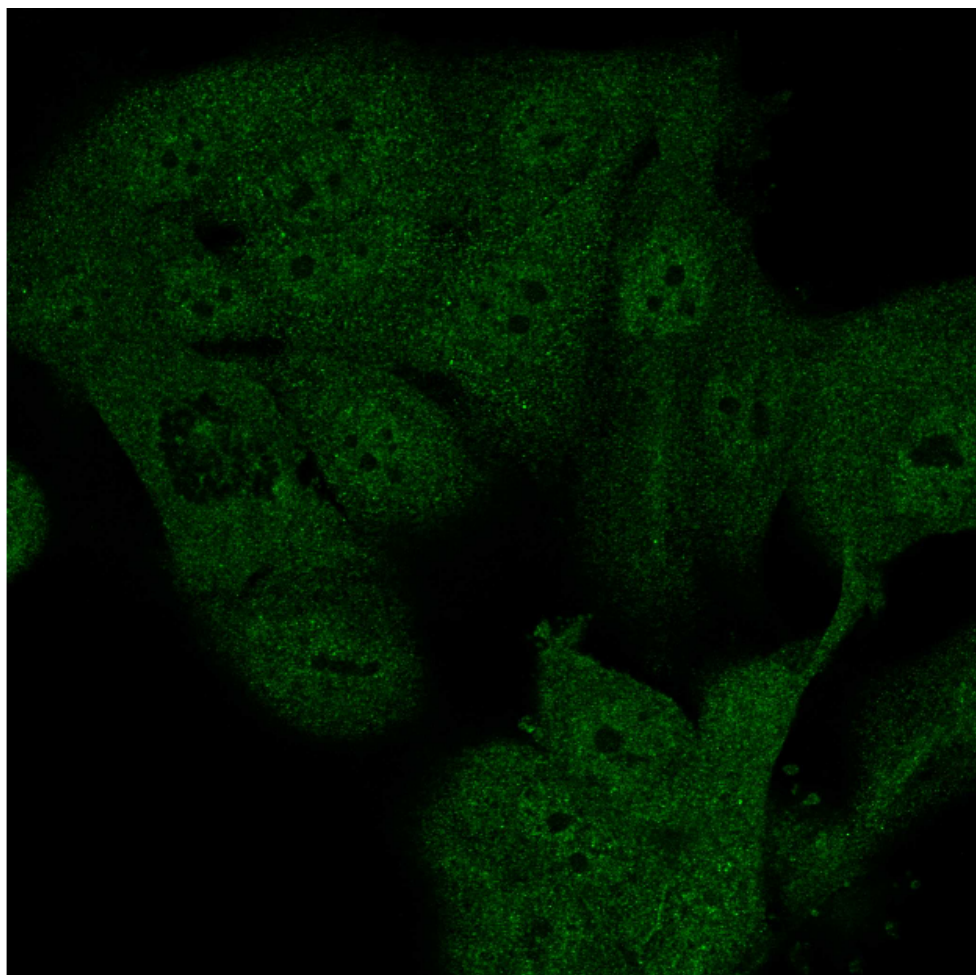

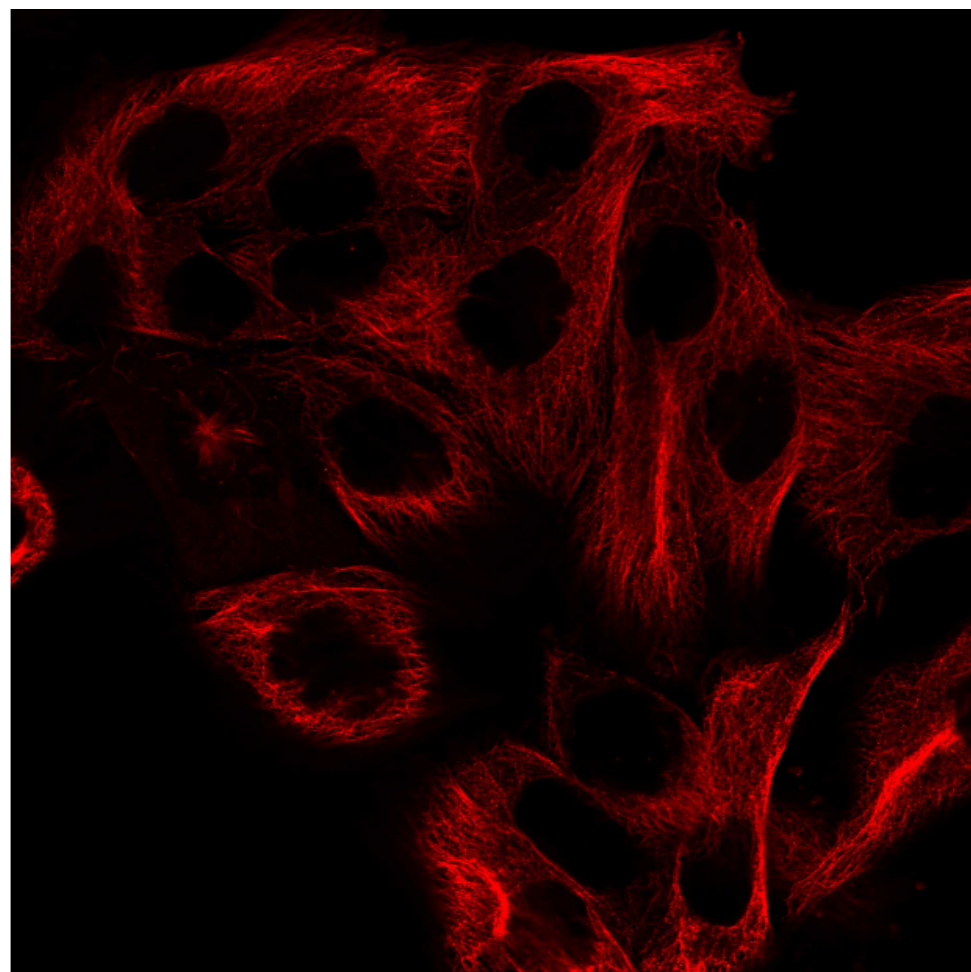

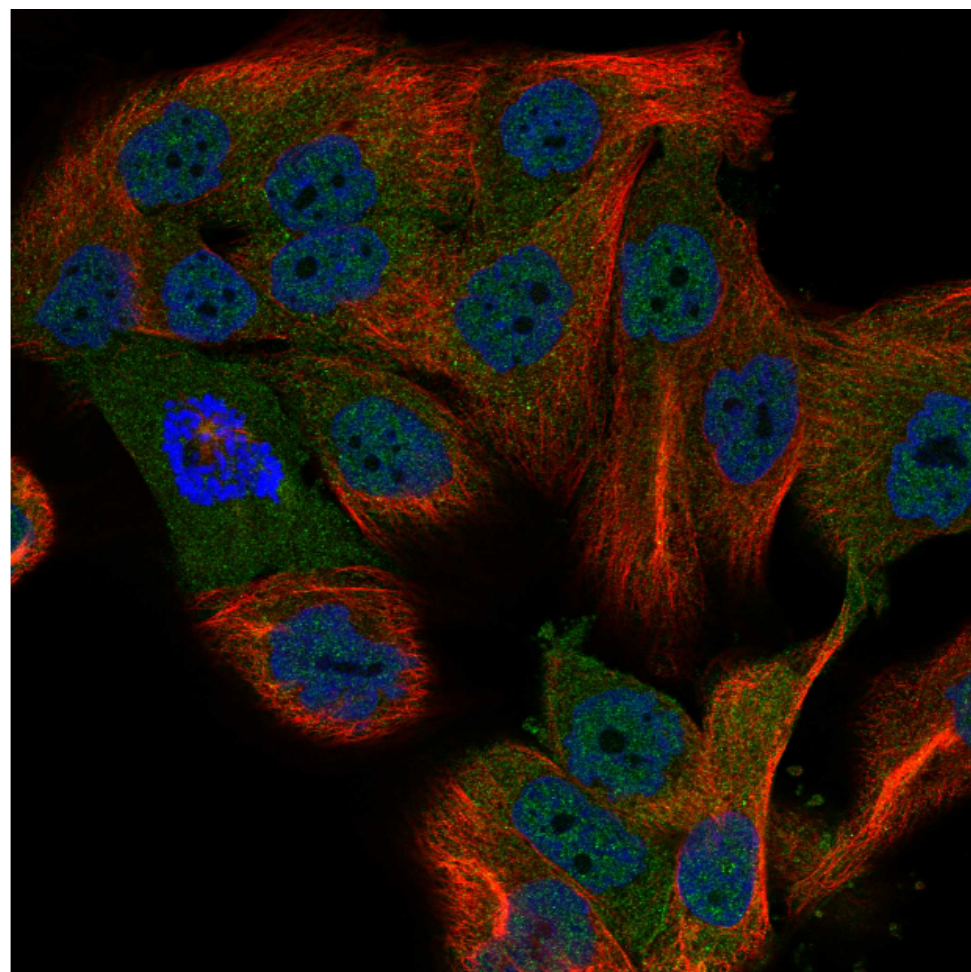

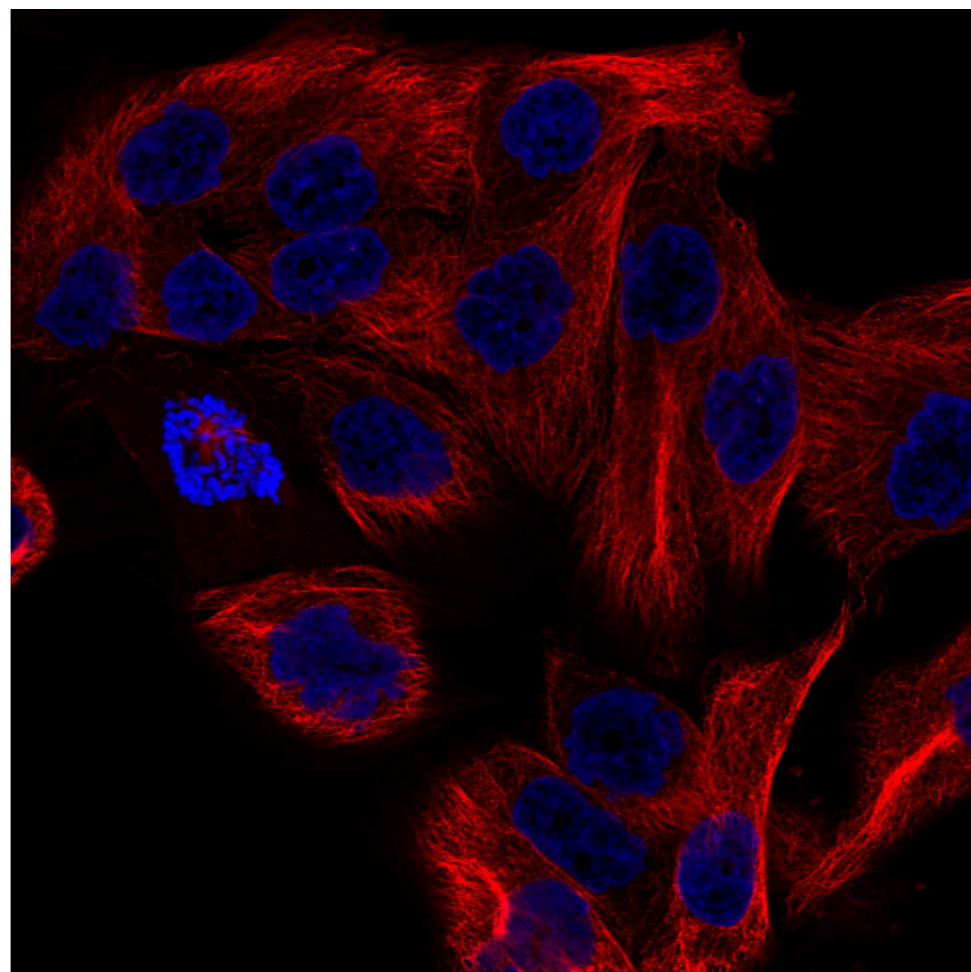

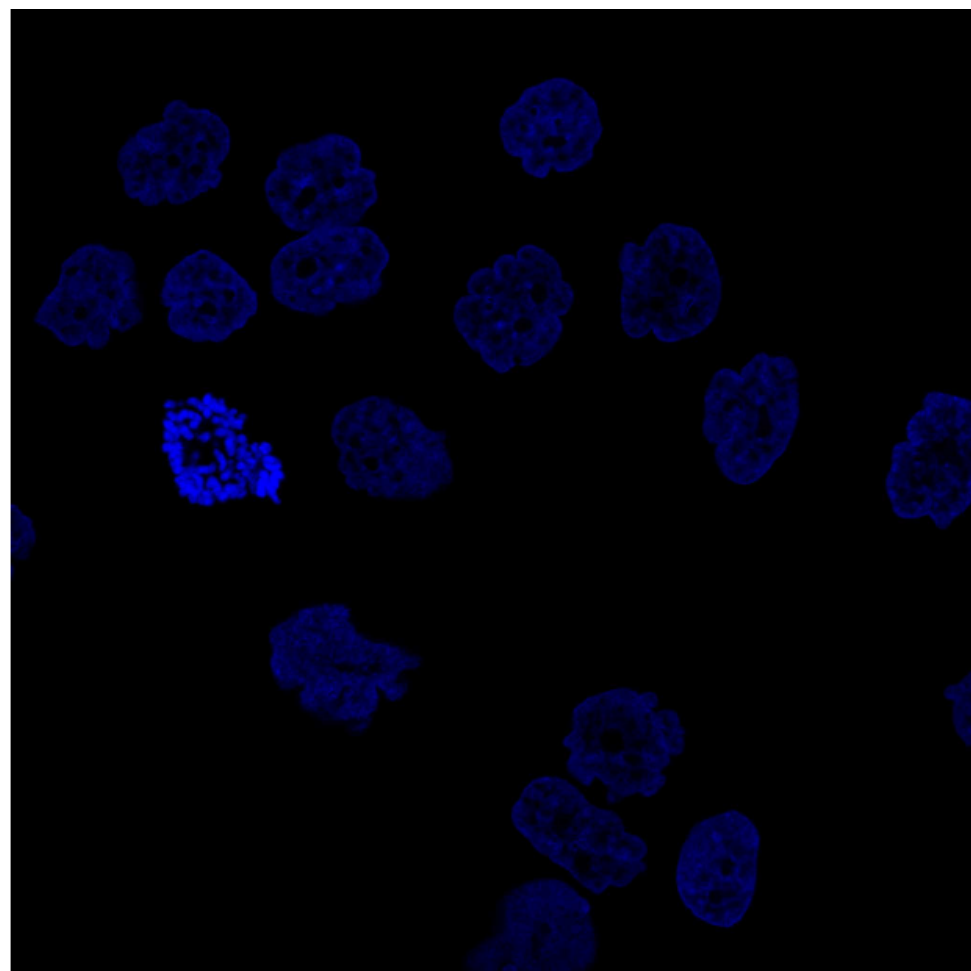

Supplement: Supplementary file 4 [file DataSheet_4.pdf]

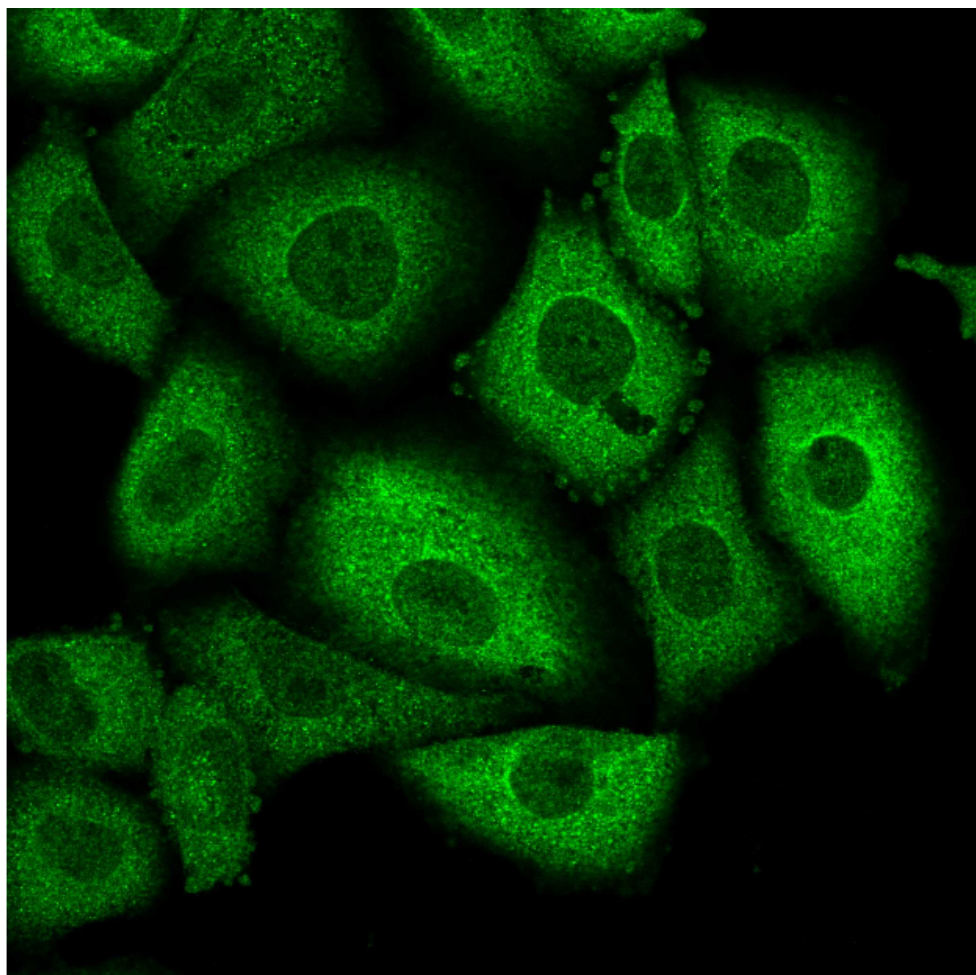

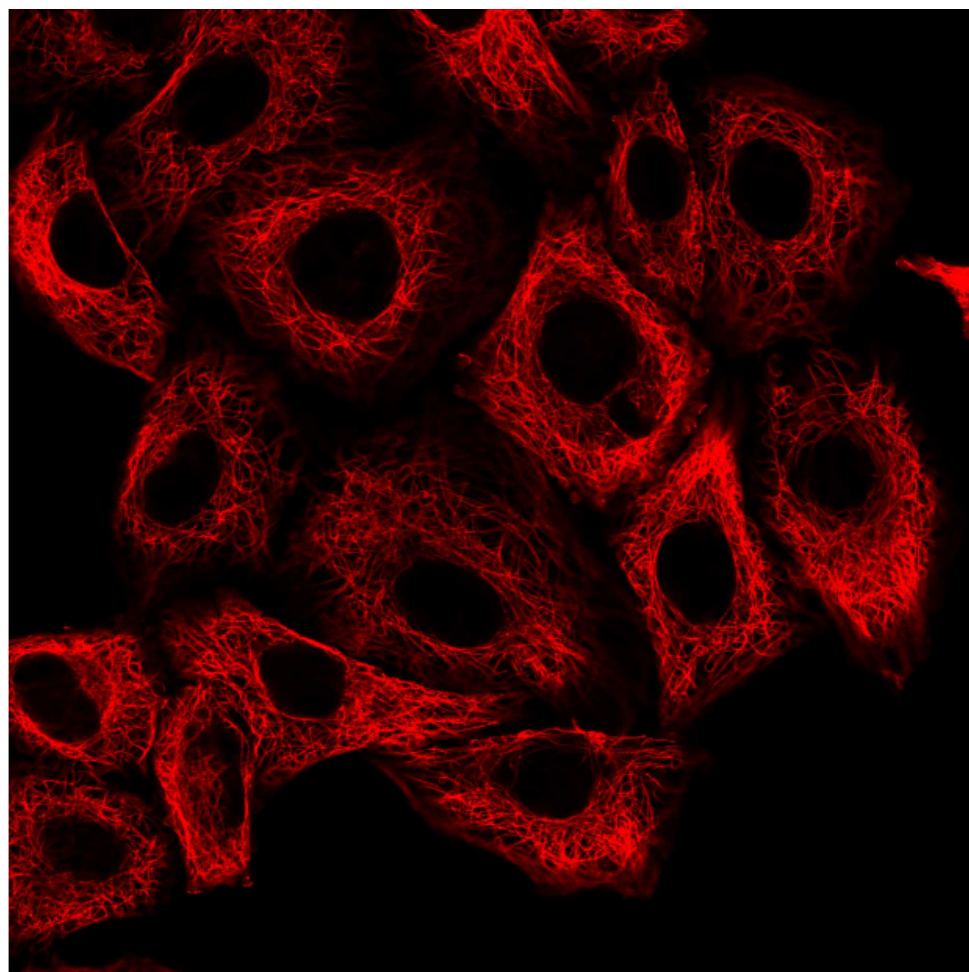

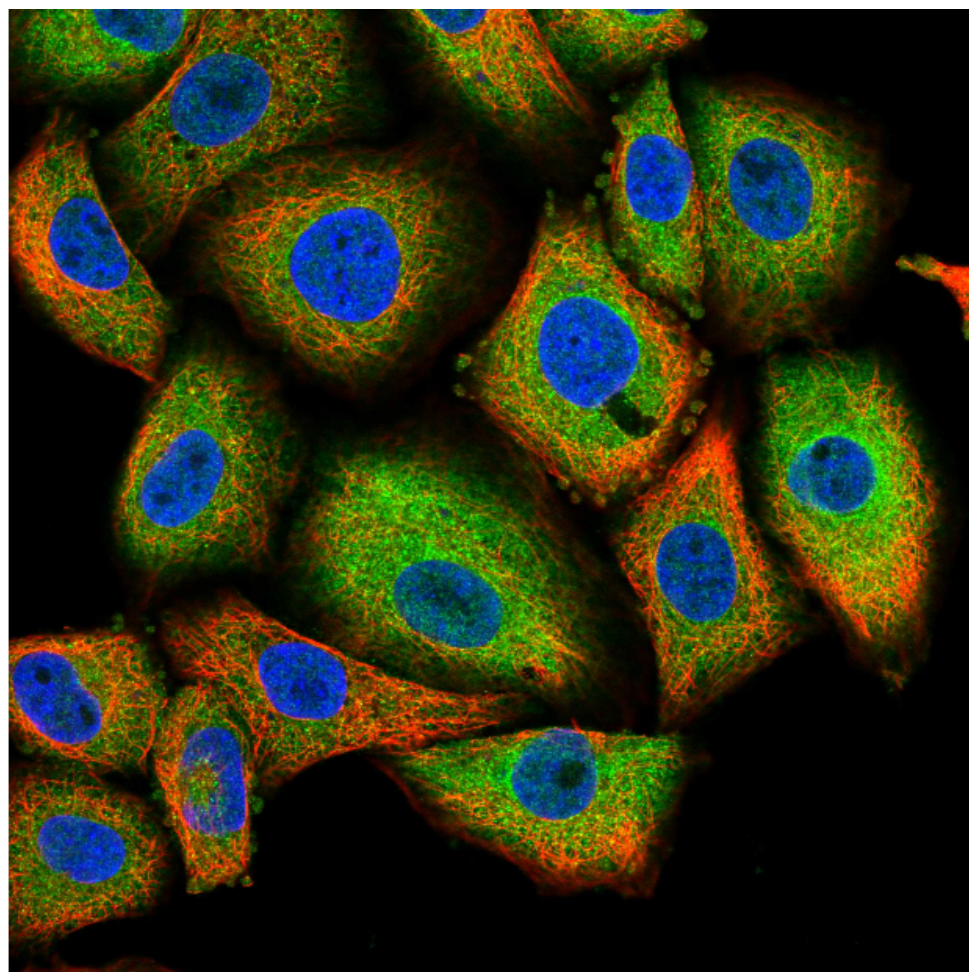

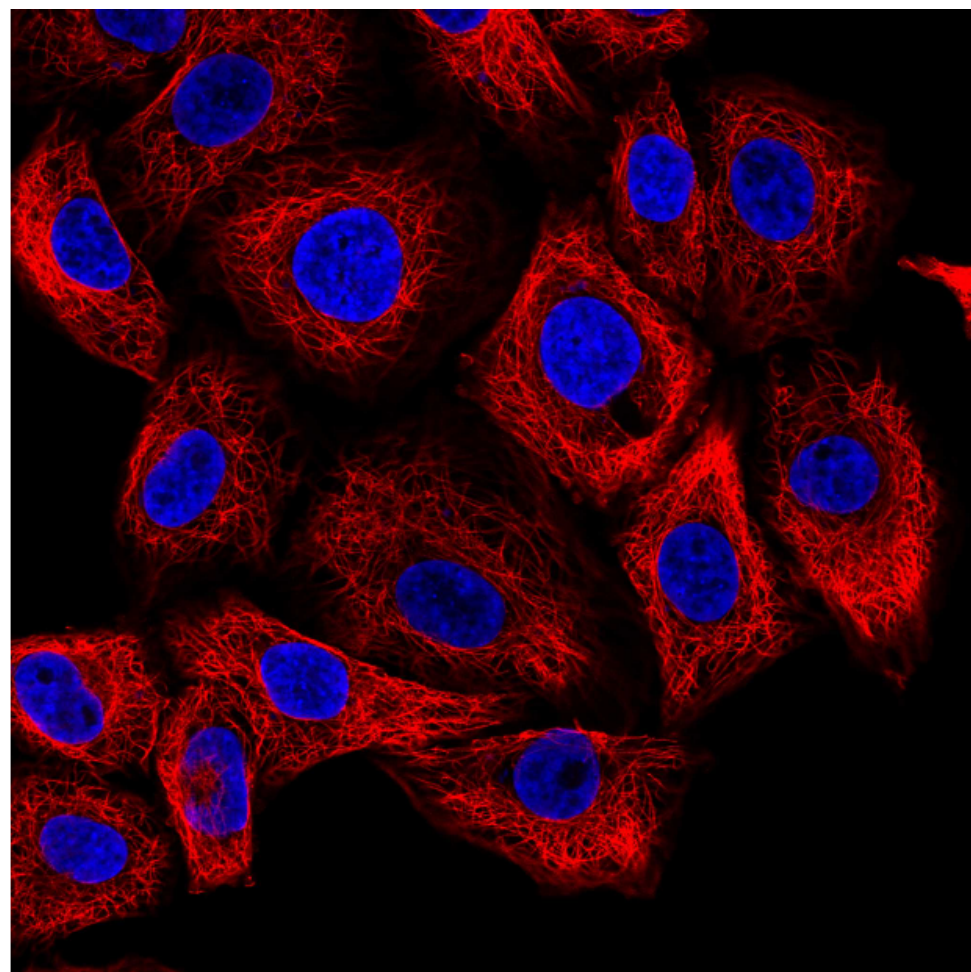

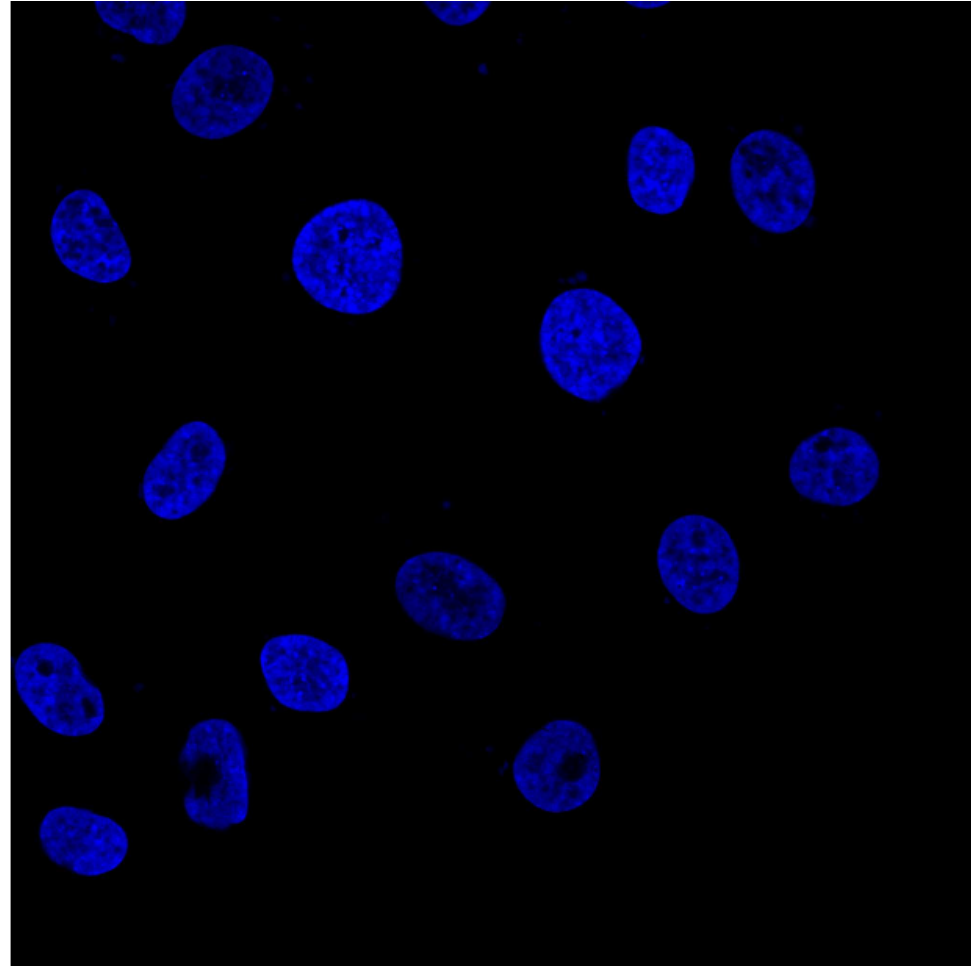

Supplement: Supplementary file 5 [file DataSheet_5.pdf]

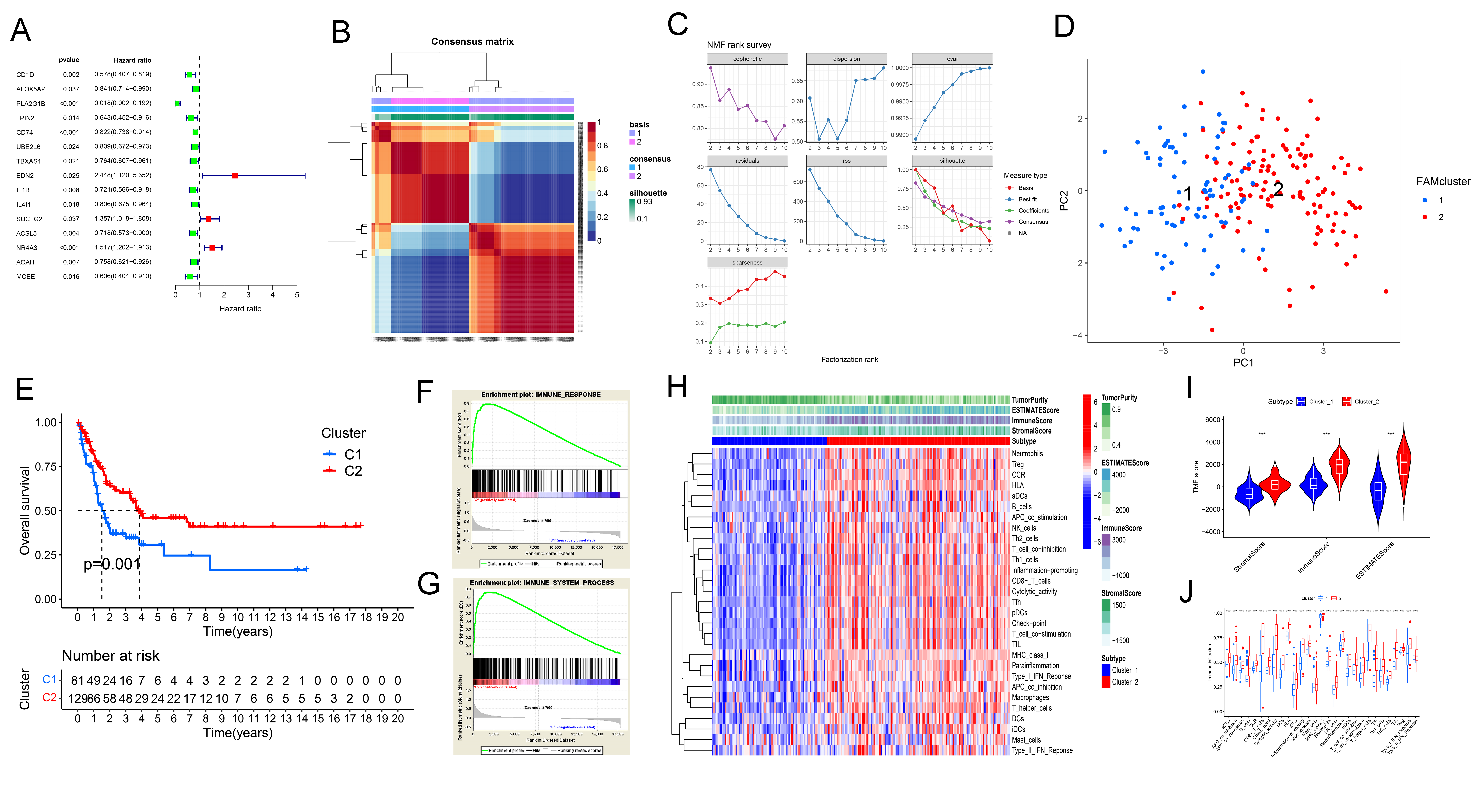

Supplement: Supplementary file 6 [file Image_1.tif]

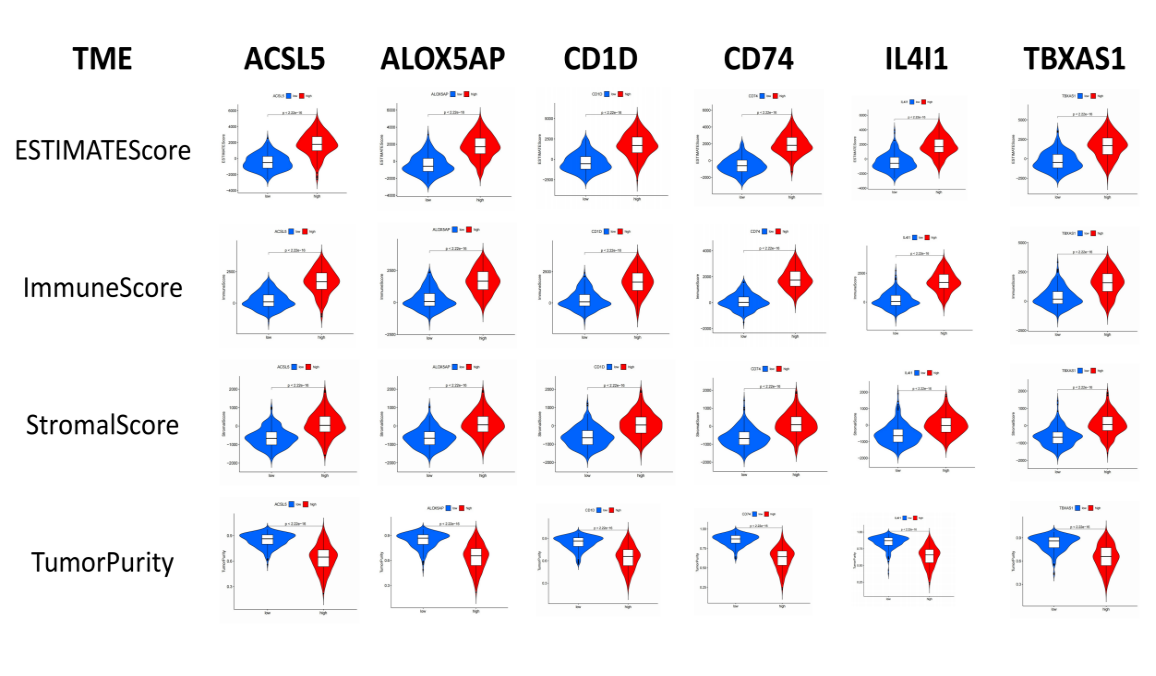

Supplement: Supplementary file 7 [file Image_2.tif]

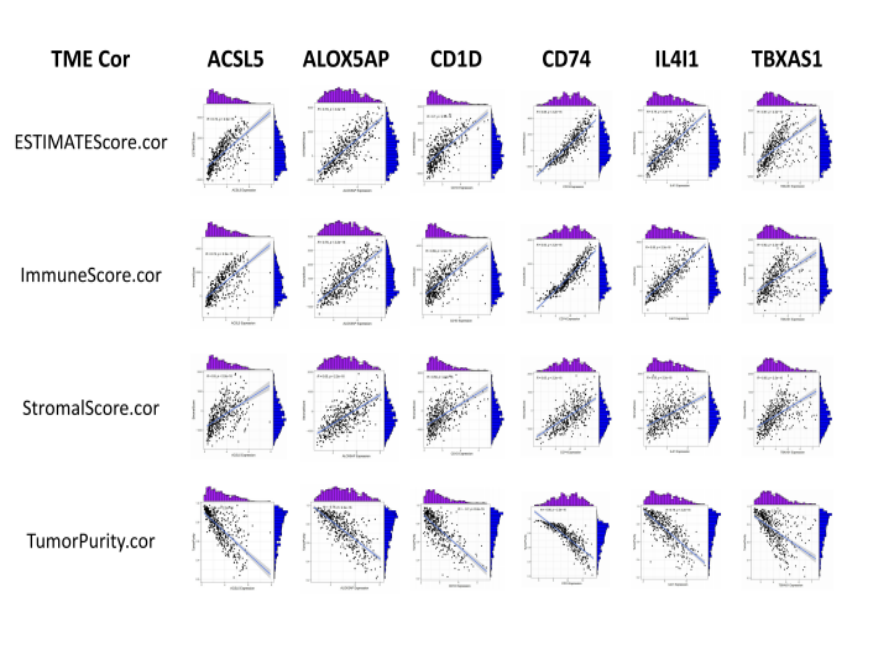

Supplement: Supplementary file 8 [file Image_3.tif]

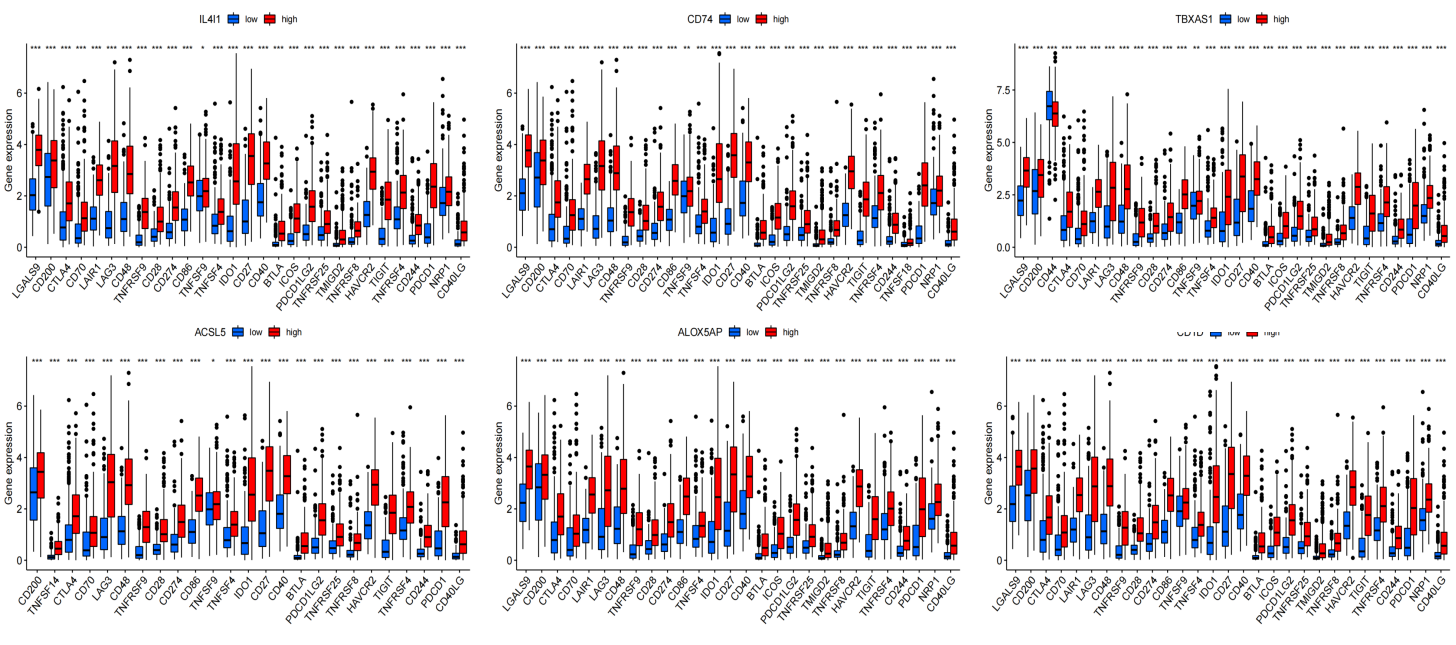

Supplement: Supplementary file 9 [file Image_4.tif]
